# Electromechanical Modeling of Vibration-Based Piezoelectric Nanogenerator with Multilayered Cross-Section for Low-Power Consumption Devices

**DOI:** 10.3390/mi11090860

**Published:** 2020-09-17

**Authors:** Ernesto A. Elvira-Hernández, Juan C. Anaya-Zavaleta, Eustaquio Martínez-Cisneros, Francisco López-Huerta, Luz Antonio Aguilera-Cortés, Agustín L. Herrera-May

**Affiliations:** 1 Micro and Nanotechnology Research Center, Universidad Veracruzana, Calzada Ruiz Cortines 455, Boca del Río, Veracruz 94294, Mexico; aelvirah@hotmail.com (E.A.E.-H.); zs17024416@estudiantes.uv.mx (E.M.-C.); 2 Departamento de Ingeniería Mecánica, DICIS, Universidad de Guanajuato, Carretera Salamanca-Valle de Santiago km 3.5 + 1.8, Salamanca 36885, Mexico; jc.anayazavaleta@ugto.mx (J.C.A.-Z.); aguilera@ugto.mx (L.A.A.-C.); 3 Facultad de Ingeniería Eléctrica y Electrónica, Universidad Veracruzana, Calzada Ruíz Cortines 455, Boca del Río, Veracruz 94294, Mexico; frlopez@uv.mx; 4 Maestría en Ingeniería Aplicada, Facultad de Ingeniería de la Construcción y el Hábitat, Universidad Veracruzana, Calzada Ruíz Cortines 455, Boca del Río, Veracruz 94294, Mexico

**Keywords:** double-clamped multilayered beam, bending resonant frequency, Euler–Bernoulli beam theory, Macaulay method, piezoelectric nanogenerator, Rayleigh method

## Abstract

Piezoelectric nanogenerators can convert energy from ambient vibrations into electrical energy. In the future, these nanogenerators could substitute conventional electrochemical batteries to supply electrical energy to consumer electronics. The optimal design of nanogenerators is fundamental in order to achieve their best electromechanical behavior. We present the analytical electromechanical modeling of a vibration-based piezoelectric nanogenerator composed of a double-clamped beam with five multilayered cross-sections. This nanogenerator design has a central seismic mass (910 μm thickness) and substrate (125 μm thickness) of polyethylene terephthalate (PET) as well as a zinc oxide film (100 nm thickness) at the bottom of each end. The zinc oxide (ZnO) films have two aluminum electrodes (100 nm thickness) through which the generated electrical energy is extracted. The analytical electromechanical modeling is based on the Rayleigh method, Euler–Bernoulli beam theory and Macaulay method. In addition, finite element method (FEM) models are developed to estimate the electromechanical behavior of the nanogenerator. These FEM models consider air damping at atmospheric pressure and optimum load resistance. The analytical modeling results agree well with respect to those of FEM models. For applications under accelerations in *y*-direction of 2.50 m/s^2^ and an optimal load resistance of 32,458 Ω, the maximum output power and output power density of the nanogenerator at resonance (119.9 Hz) are 50.44 μW and 82.36 W/m^3^, respectively. This nanogenerator could be used to convert the ambient mechanical vibrations into electrical energy and supply low-power consumption devices.

## 1. Introduction

The world population growth and technological development have increased the energy consumption through electronic components and devices. For instance, the world population will use approximately 25 billion devices in the following years [1]. These devices will need sources of electrical energy that do not cause environmental contamination. For this, the conventional electrochemical batteries are not suitable due to their toxic materials that may generate ambient damage. In addition, the electrochemical batteries have large volume, which may increase the size of the electronic devices. Additionally, these batteries have short operating times that interrupt the supply of electrical energy to the devices. In order to substitute these batteries, the micro and nanogenerators can be employed to obtain electrical energy from ambient vibrations or mechanical motions such as vehicles vibrations, human body motions, buildings vibrations, water wave motions, and wind sources [2,3,4,5,6,7,8,9,10,11]. These micro and nanogenerators can use different transduction mechanisms: electromagnetic, electrostatic, triboelectric, thermoelectric or piezoelectric [12,13,14,15,16,17,18,19,20,21,22,23,24,25]. In comparison with electrochemical batteries, the micro and nanogenerators have advantages such as sustainability, renewability and flexibility [26]. These devices can use specific conditioning circuits as diode-bridge rectifiers to charge capacitors to direct current (DC) voltage. In the design phase, the performance of the micro and nanogenerators can be estimated using analytical electromechanical modeling. By using this modeling, the designers can determine the better geometrical configurations, materials and dimensions of the micro and nanogenerators for each specific application. Furthermore, the output voltages of the micro and nanogenerators can be increased when they are designed to operate at resonance with similar frequencies to those of ambient vibrations or mechanical motions. Thus, the values of main design parameters of the micro and nanogenerators can be modified using analytical electromechanical modeling to increase their output voltage and power. However, small variations in the different layers and cross-sections of the micro and nanogenerators can affect their performance at resonance. To obtain better estimations about the performance of these devices, the analytical electromechanical modeling should consider all their layers and variations along the cross-sections.

Commonly, the piezoelectric micro and nanogenerators have been investigated to convert kinetic energy from vibrations or mechanical motions into electrical energy [27,28,29,30,31,32,33,34]. This is due to the simple fabrication process, ease of application, and high power density of the piezoelectric materials [35,36]. In order to increase the power density of these devices, their structures could be designed to operate at resonance. For this, the fundamental resonant frequency of the micro and nanogenerators must match with that of ambient vibrations or mechanical motions. Thus, the development of electromechanical models for nanogenerators are required to determine their best geometrical configurations and materials. Some researchers have reported analytical electromechanical models for piezoelectric micro and nanogenerators [37,38,39,40]. Martínez-Cisneros et al. [37] presented the analytical electromechanical modeling of a piezoelectric microgenerator with T shape for applications in domestic washing machines. This modeling considers a resonator with two different multilayered cross-sections, but it is only suitable for single-clamped beams. In addition, Elvira-Hernández et al. [38] registered the analytical electromechanical modeling of a piezoelectric microgenerator with three different multilayered cross-sections for air conditioning vents in office buildings. Although, this modeling is applied to a microgenerator based on single-clamped beam. On the other hand, Qin et al. [39] reported an analytical electromechanical coupling model of a bridge-shaped piezoelectric microgenerator using the two-dimensional nonlinear Euler–Bernoulli beam theory and Hamilton principle. This model adequately predicts the voltage generated by the device; however, it is limited to uniform cross-sections with two layers and a seismic mass. Abdelkefi and Barsallo [40] established the analytical electromechanical model for a piezoelectric-magnetoelastic microgenerator formed by a single-clamped beam with a magnet mass located on the beam tip. However, this model is only suitable for single-clamped beams with two uniform cross-sections and two layers. Here, we propose the analytical electromechanical modeling of a piezoelectric nanogenerator formed by a double-clamped beam with five cross-sections, including its flexible substrate, two electrodes, a piezoelectric film, and a seismic mass. This model was obtained using the Rayleigh method, the Euler–Bernoulli beam theory and the Macaulay method. Additionally, the quality factor of the nanogenerator due to the air damping is calculated. Based on the electromechanical model, the dimensions of the piezoelectric nanogenerator can be adjusted to modify its first bending resonant frequency, deflections, and output power. Thus, the best dimensions of the nanogenerator can be selected to improve its performance for each potential application. The nanogenerator is designed with a flexible substrate (125 μm thickness) of polyethylene terephthalate (PET), two electrodes (100 nm thickness) of aluminum, a piezoelectric film (100 nm thick) of zinc oxide (ZnO), and a central seismic mass (910 μm thickness). Moreover, finite element method (FEM) models of the nanogenerator are developed to predict its electromechanical performance. This device at resonance (119.9 Hz) can generate a maximum output power of 50.44 μW under acceleration in *y*-direction of 2.50 m/s^2^, and an optimal load resistance of 32,458 Ω. An array of these nanogenerators with diode-bridge rectifiers can be used to charge capacitors to DC voltage. This electrical charge could supply low-power consumption electronic devices.

This work is organized as follows: Section 2 includes the description of the analytical and FEM models of the piezoelectric nanogenerator to determine its first bending resonant frequency, out-plane displacements and output voltage. Section 3 depicts the results and discussions of the analytical and FEM models. Finally, Section 4 reports the conclusions and future research work.

## 2. Analytical Modeling of the Nanogenerator

In this section, we present the modeling to estimate the electromechanical behavior of the piezoelectric nanogenerator. In addition, the quality factor and air damping at atmospheric pressure of the nanogenerator are obtained.

### 2.1. Design

**Figure 1:**
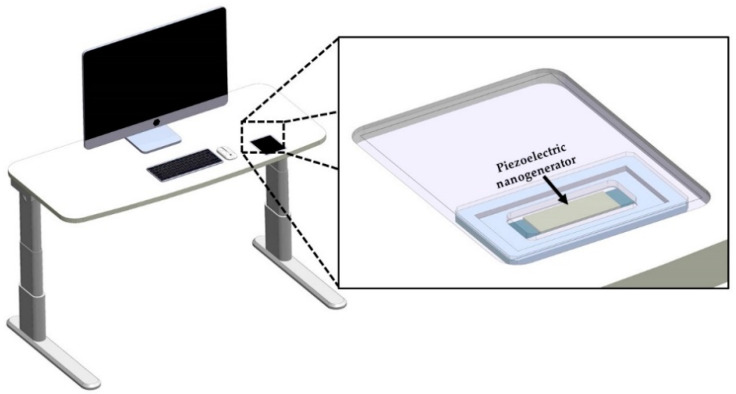
Isometric view of a piezoelectric nanogenerator located at the bottom side of an electronic display at a smart office desk.

**Figure 2:**
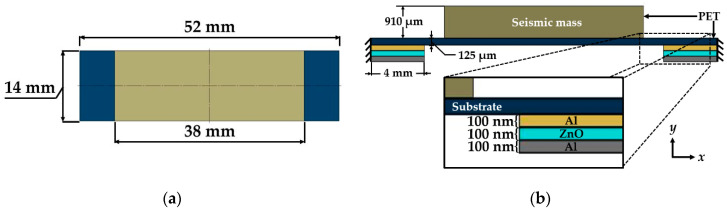
(**a**) Top view and (**b**) front side view (not to scale) of the different layers of the piezoelectric nanogenerator.

A piezoelectric nanogenerator (Figure 1) is designed to convert the energy from mechanical vibrations into electrical energy. For instance, the mechanical vibrations of office desks (120 Hz) [41] could be transformed into electrical energy using the proposed nanogenerator. Thus, an array of nanogenerators with diode-bridge rectifiers could charge capacitors to DC voltage. This voltage could supply low-power consumption devices. This nanogenerator can be collocated on the bottom side of office desks (e.g., bottom side on an electronic display at smart office desks). The proposed nanogenerator has a double-clamped beam with five cross-sections, as shown in Figure 2a,b. A double-clamped beam with a central seismic mass is selected to take advantage of two stress concentration surfaces generated near both fixed ends of the beam [42], in where the piezoelectric layers are located. In addition, the double-clamped beams can provide a better stable and reliable operation than cantilevered structures [43]. This beam includes a PET flexible substrate (52 mm × 14 mm × 125 µm) with a PET seismic mass (38 mm × 14 mm × 910 µm), a ZnO layer (4 mm × 14 mm × 100 nm) located on each end of the beam, and two aluminum electrodes (4 mm × 14 mm × 100 nm) between each ZnO layer. The aluminum electrodes are chosen due to their good adherence with the PET and ZnO layers, which can decrease the delamination of these layers during the nanogenerator operation [44]. ZnO layers do not contain toxic materials and they can undergo large deformations for long periods. In addition, ZnO layers do not require a subsequent polarization process [45,46].

**Figure 3:**
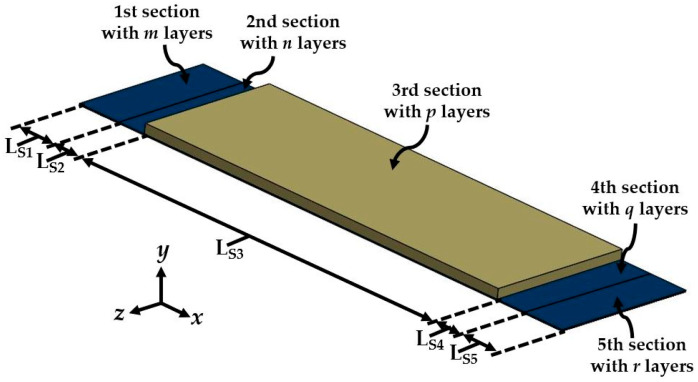
Schematic view of the piezoelectric nanogenerator with five multilayered sections.

**Figure 4:**
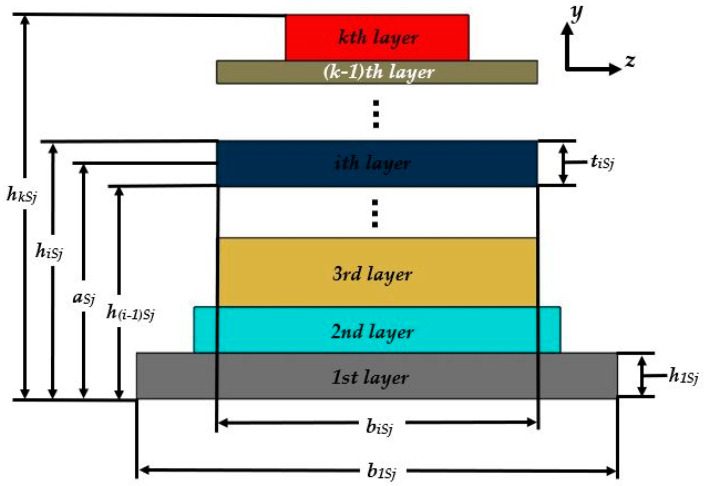
Schematic view of the layers located on the *j*th section of the piezoelectric nanogenerator.

The Rayleigh method [47,48] is used to estimate the first bending resonant frequency of the double-clamped beam with multilayered cross-sections of the nanogenerator. The device is divided into five sections with a different number of layers (Figure 3). Each section has *m*th, *n*th, *p*th, *q*th and *r*th layers, which have symmetry on the *x-y* plane. Figure 4 depicts a schematic view and nomenclature of the different layers of the *j*th cross-section of the nanogenerator. In order to simplify the analytical model, the layers of the nanogenerator are assumed as homogeneous and isotropic.

The elastic centroid (*a_Sj_*) of the *j*th section of the piezoelectric nanogenerator can be calculated as [49]:

(1) $$a_{Sj}=\frac{{\left(ES\right)}_{Sj}}{{\left(EA\right)}_{Sj}}=\frac{\int \int_{A_{Sj}}E_{Sj}y_{Sj}(x)dydz}{\int \int_{A_{Sj}}E_{Sj}dydz}=\frac{1}{2}\frac{\sum_{i=1}^{k}E_{iSj}b_{iSj}t_{iSj}\left[\left(h_{iSj}+h_{(i-1)Sj}\right)\right]}{\sum_{i=1}^{q}E_{iSj}b_{iSj}t_{iSj}}$$

where *E_iSj_* is Young’s modulus of the *i*th layer in the *j*th section, *h*_(*i*−1)*Sj*_ is the distance from bottom side of the first layer to the top side of the (*i* − 1)th layer of the *j*th section, *h*_iSj_ is the distance from the bottom side of the first layer to the top face of the *i*th layer of the *j*th section, *b_iSj_* and *t_iSj_* = *h_iSj_* − *h*_(*i*−1)*Sj*_ are the width and thickness of the *i*th layer located in the *j*th section. The parameter *k* is the number of layers in each one of the five sections (*k* = *m*, *n*, *p*, *q*, *r*) and *A_Sj_* is the area of the *j*th section.

The elastic centroid of each section is used to calculate its bending stiffness (*EI_z_*)*_Sj_* as [50]:

(2) $${\left(EI_{z}\right)}_{Sj}=\sum_{i=1}^{k}{\left(E_{i}I_{zi}\right)}_{Sj}=\int \int_{A_{Sj}}E_{Sj}y_{Sj}(x)dy=\frac{1}{3}\sum_{i=1}^{k}E_{iSj}b_{iSj}\left[{\left(h_{iSj}-a_{Sj}\right)}^{3}-{\left(h_{(i-1)Sj}-a_{Sj}\right)}^{3}\right]$$

Based on the Rayleigh method, the maximum kinetic (*K_m_*) and potential (*P_m_*) energies of the five sections are determined as:

(3) $$\begin{array}{ll} P_{m}= & \;\frac{1}{2}({\left(EI_{z}\right)}_{S_{1}}{\int_{0}^{L_{1}}\left(\frac{d^{2}y_{S_{1}}(x)}{dx^{2}}\right)}^{2}\;dx+{\left(EI_{z}\right)}_{S_{2}}{\int_{L_{1}}^{L_{12}}\left(\frac{d^{2}y_{S_{2}}(x)}{dx^{2}}\right)}^{2}\;dx\\ & +{\left(EI_{z}\right)}_{S_{3}}{\int_{L_{12}}^{L_{123}}\left(\frac{d^{2}y_{S_{3}}(x)}{dx^{2}}\right)}^{2}\;dx+{\left(EI_{z}\right)}_{S_{4}}{\int_{L_{123}}^{L_{1234}}\left(\frac{d^{2}y_{S_{4}}(x)}{dx^{2}}\right)}^{2}\;dx\\ & +{\left(EI_{z}\right)}_{S_{5}}{\int_{L_{1234}}^{L_{12345}}\left(\frac{d^{2}y_{S_{5}}(x)}{dx^{2}}\right)}^{2}\;dx) \end{array}$$

(4) $$\begin{array}{ll} K_{m}= & \frac{1}{2}(\left(\sum_{i=1}^{m}\rho_{iS_{1}}b_{iS_{1}}t_{iS_{1}}\right)\int_{0}^{L_{1}}{\left(y_{S_{1}}(x)\right)}^{2}dx+\left(\sum_{i=1}^{n}\rho_{iS_{2}}b_{iS_{2}}t_{iS_{2}}\right)\int_{L_{1}}^{L_{12}}{\left(y_{S_{2}}(x)\right)}^{2}dx\\ & +\left(\sum_{i=1}^{p}\rho_{iS_{3}}b_{iS_{3}}t_{iS_{3}}\right)\int_{L_{12}}^{L_{123}}{\left(y_{S_{3}}(x)\right)}^{2}dx+\left(\sum_{i=1}^{q}\rho_{iS_{4}}b_{iS_{4}}t_{iS_{4}}\right)\int_{L_{123}}^{L_{1234}}{\left(y_{S_{4}}(x)\right)}^{2}dx\\ & +\left(\sum_{i=1}^{r}\rho_{iS_{5}}b_{iS_{5}}t_{iS_{5}}\right)\int_{L_{1234}}^{L_{12345}}{\left(y_{S_{5}}(x)\right)}^{2}dx) \end{array}$$

where *L*_12_ = *L*_1_ + *L*_2_, *L*_123_ = *L*_1_ + *L*_2_ + *L*_3_, *L*_1234_ = *L*_1_ + *L*_2_ + *L*_3_ + *L*_4_ and *L*_12345_ = *L*_1_ + *L*_2_ + *L*_3_ + *L*_4_ + *L*_5_. Furthermore, *y_Sj_* is the static deflection of the *j*th section and *ρ_iSj_* is the density of the *i*th layer in the *j*th section.

Applying the conservation of energy (*P_m_* = *K_m_*), we obtain the resonance frequency as:

(5) $$f_{r}=\frac{1}{2\pi }\sqrt{\frac{P_{m}}{K_{m}}}$$

The Equations (3) and (4) need the values of deflections (*y_Sj_*) of the five sections of nanogenerator. We consider an initial deflection of the nanogenerator equal to zero. After, the deflections of the nanogenerator can be calculated using the Euler–Bernoulli beam theory [51]:

(6) $${\left(EI_{z}\right)}_{S_{1}}\frac{\partial^{2}y_{S_{1}}(x)}{\partial x^{2}}=M_{S_{1}}(x),\;0<x<L_{1}$$

(7) $${\left(EI_{z}\right)}_{S_{2}}\frac{\partial^{2}y_{S_{2}}(x)}{\partial x^{2}}=M_{S_{2}}(x),\;L_{1}<x<L_{12}$$

(8) $${\left(EI_{z}\right)}_{S_{3}}\frac{\partial^{2}y_{S_{3}}(x)}{\partial x^{2}}=M_{S_{3}}(x),\;L_{12}<x<L_{123}$$

(9) $${\left(EI_{z}\right)}_{S_{4}}\frac{\partial^{2}y_{S_{4}}(x)}{\partial x^{2}}=M_{S_{4}}(x),\;L_{123}<x<L_{1234}$$

(10) $${\left(EI_{z}\right)}_{S_{5}}\frac{\partial^{2}y_{S_{5}}(x)}{\partial x^{2}}=M_{S_{5}}(x),\;L_{1234}<x<L_{12345}$$

where *M_Sj_* is the bending moment of the *j*th section.

The boundary conditions for each one of the sections of the nanogenerator are given by:

(11) $$y_{1}\left(0\right)=0,\;\frac{\partial y_{1}\left(0\right)}{\partial x}=0$$

(12) $$y_{1}\left(L_{1}\right)=y_{2}\left(L_{1}\right),\;\frac{\partial y_{1}\left(L_{1}\right)}{\partial x}=\frac{\partial y_{2}\left(L_{1}\right)}{\partial x}$$

(13) $$y_{2}\left(L_{12}\right)=y_{3}\left(L_{12}\right),\;\frac{\partial y_{2}\left(L_{12}\right)}{\partial x}=\frac{\partial y_{3}\left(L_{12}\right)}{\partial x}$$

(14) $$y_{3}\left(L_{123}\right)=y_{4}\left(L_{123}\right),\;\frac{\partial y_{3}\left(L_{123}\right)}{\partial x}=\frac{\partial y_{4}\left(L_{123}\right)}{\partial x}$$

(15) $$y_{4}\left(L_{1234}\right)=y_{5}\left(L_{1234}\right),\;\frac{\partial y_{4}\left(L_{1234}\right)}{\partial x}=\frac{\partial y_{5}\left(L_{1234}\right)}{\partial x}$$

The bending moment is determined by integrating twice the load function (*F* (*x*)) and applying the Macaulay method [52].

(16) $$\begin{array}{ll} F(x)= & -M_{0}{\langle x-0\rangle }^{-2}+R_{0}{\langle x-0\rangle }^{-1}-\omega_{S_{1}}{\langle x-0\rangle }^{0}+\omega_{S_{1}}{\langle x-L_{1}\rangle }^{0}-\omega_{S_{2}}{\langle x-L_{1}\rangle }^{0}\\ & +\omega_{S_{2}}{\langle x-L_{12}\rangle }^{0}-\omega_{S_{3}}{\langle x-L_{12}\rangle }^{0}+\omega_{S_{3}}{\langle x-L_{123}\rangle }^{0}-\omega_{S_{4}}{\langle x-L_{123}\rangle }^{0}\\ & +\omega_{S_{4}}{\langle x-L_{1234}\rangle }^{0}-\omega_{S_{5}}{\langle x-L_{1234}\rangle }^{0}+\omega_{S_{5}}{\langle x-L_{12345}\rangle }^{0}+R_{1}{\langle x-L_{12345}\rangle }^{-1}\\ & +M_{1}{\langle x-L_{12345}\rangle }^{-2} \end{array}$$

Considering the integration rules of the Macaulay functions, the shear load function *V*(*x*) is obtained by integrating Equation (16):

(17) $$\begin{array}{ll} V(x)= & -M_{0}{\langle x-0\rangle }^{-1}+R_{0}{\langle x-0\rangle }^{0}-\omega_{S_{1}}{\langle x-0\rangle }^{1}+\omega_{S_{1}}{\langle x-L_{1}\rangle }^{1}-\omega_{S_{2}}{\langle x-L_{1}\rangle }^{1}\\ & +\omega_{S_{2}}{\langle x-L_{12}\rangle }^{1}-\omega_{S_{3}}{\langle x-L_{12}\rangle }^{1}+\omega_{S_{3}}{\langle x-L_{123}\rangle }^{1}-\omega_{S_{4}}{\langle x-L_{123}\rangle }^{1}+\omega_{S_{4}}{\langle x-L_{1234}\rangle }^{1}\\ & -\omega_{S_{5}}{\langle x-L_{1234}\rangle }^{1}+\omega_{S_{5}}{\langle x-L_{12345}\rangle }^{1}+R_{1}{\langle x-L_{12345}\rangle }^{0}+M_{1}{\langle x-L_{12345}\rangle }^{-1}+C_{1} \end{array}$$

Next, bending moment function *M(x)* is calculated by integrating the Equation (17):

(18) $$\begin{array}{ll} M(x)= & -M_{o}{\langle x-0\rangle }^{0}+R_{1}{\langle x-0\rangle }^{1}-\frac{1}{2}\omega_{S_{1}}{\langle x-0\rangle }^{2}+\frac{1}{2}\omega_{S_{1}}{\langle x-L_{1}\rangle }^{2}-\frac{1}{2}\omega_{S_{2}}{\langle x-L_{1}\rangle }^{2}\\ & +\frac{1}{2}\omega_{S_{2}}{\langle x-L_{12}\rangle }^{2}-\frac{1}{2}\omega_{S_{3}}{\langle x-L_{12}\rangle }^{2}+\frac{1}{2}\omega_{S_{3}}{\langle x-L_{123}\rangle }^{2}-\frac{1}{2}\omega_{S_{4}}{\langle x-L_{123}\rangle }^{2}\\ & +\frac{1}{2}\omega_{S_{4}}{\langle x-L_{1234}\rangle }^{2}-\frac{1}{2}\omega_{S_{5}}{\langle x-L_{1234}\rangle }^{2}+\frac{1}{2}\omega_{S_{5}}{\langle x-L_{12345}\rangle }^{2}+R_{1}{\langle x-L_{12345}\rangle }^{1}\\ & +M_{1}{\langle x-L_{12345}\rangle }^{0}+C_{1}x+C_{2} \end{array}$$

The integration constants (*C*_1_ = 0 and *C*_2_ = 0) of Equations (17) and (18) are defined with the boundary conditions *V*(0) = *R*_0_ and *M*(0) = *M*_0_.

**Figure 5:**
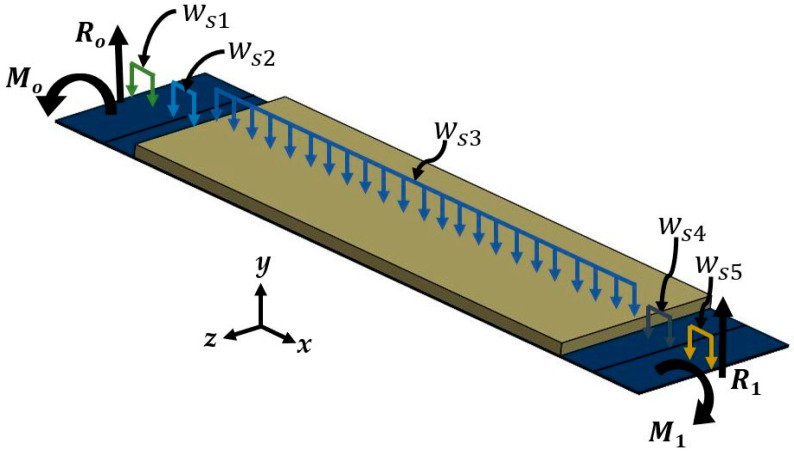
Schematic view of the different load types of the nanogenerator.

Due to the symmetry of the nanogenerator with respect to *y-z* plane (Figure 5), their bending moment and reaction force in each clamped support have the same value (*M*_0_ = *M*_1_ and *R*_0_ = *R*_1_). Moreover, the weight per unit length of sections 1 and 2 are equal to those of sections 5 and 4 (*ω_S_*_1_ = *ω_S_*_5_ and *ω_S_*_2_ = *ω_S_*_4_), respectively. To determine the reaction force (*R*_0_) on a clamped support and the weight per unit length (*ω_Sj_*) of all the sections of the nanogenerator, we use the following Equations:

(19) $$R_{0}=\sum_{i=1}^{5}\frac{1}{2}\omega_{Sj}L_{Sj}$$

(20) $$\omega_{S_{j}}=\sum_{i=1}^{5}\rho_{iS_{j}}gb_{iS_{j}}t_{iS_{j}}$$

where *g* is the gravitational acceleration.

The bending moments of the five multilayered sections of the nanogenerator are obtained through Equation (21):

For 0 < *x* < *L*_1_

(21) $$M_{S_{1}}(x)\;=-M_{o}{\langle x-0\rangle }^{0}+R_{1}{\langle x-0\rangle }^{1}-\frac{1}{2}\omega_{S_{1}}{\langle x-0\rangle }^{2}$$

For *L*_1_ < *x* < *L*_12_

(22) $$M_{S_{2}}(x)\;=-M_{o}{\langle x-0\rangle }^{0}+R_{1}{\langle x-0\rangle }^{1}-\frac{1}{2}\omega_{S_{1}}{\langle x-0\rangle }^{2}+\frac{1}{2}\omega_{S_{1}}{\langle x-L_{1}\rangle }^{2}-\frac{1}{2}\omega_{S_{2}}{\langle x-L_{1}\rangle }^{2}$$

For *L*_12_ < *x* < *L*_123_

(23) $$\begin{array}{ll} M_{S_{3}}(x)\;= & -M_{o}{\langle x-0\rangle }^{0}+R_{1}{\langle x-0\rangle }^{1}-\frac{1}{2}\omega_{S_{1}}{\langle x-0\rangle }^{2}+\frac{1}{2}\omega_{S_{1}}{\langle x-L_{1}\rangle }^{2}-\frac{1}{2}\omega_{S_{2}}{\langle x-L_{1}\rangle }^{2}\\ & +\frac{1}{2}\omega_{S_{2}}{\langle x-L_{12}\rangle }^{2}-\frac{1}{2}\omega_{S_{3}}{\langle x-L_{12}\rangle }^{2} \end{array}$$

For *L*_123_ *< x < L*_1234_

(24) $$\begin{array}{ll} M_{S_{4}}(x)\;= & -M_{o}{\langle x-0\rangle }^{0}+R_{1}{\langle x-0\rangle }^{1}-\frac{1}{2}\omega_{S_{1}}{\langle x-0\rangle }^{2}+\frac{1}{2}\omega_{S_{1}}{\langle x-L_{1}\rangle }^{2}-\frac{1}{2}\omega_{S_{2}}{\langle x-L_{1}\rangle }^{2}\\ & +\frac{1}{2}\omega_{S_{2}}{\langle x-L_{12}\rangle }^{2}-\frac{1}{2}\omega_{S_{3}}{\langle x-L_{12}\rangle }^{2}+\frac{1}{2}\omega_{S_{3}}{\langle x-L_{123}\rangle }^{2}-\frac{1}{2}\omega_{S_{4}}{\langle x-L_{123}\rangle }^{2} \end{array}$$

For *L*_1234_ *< x < L*_12345_

(25) $$\begin{array}{ll} M_{S_{4}}(x)\;= & -M_{o}{\langle x-0\rangle }^{0}+R_{1}{\langle x-0\rangle }^{1}-\frac{1}{2}\omega_{S_{1}}{\langle x-0\rangle }^{2}+\frac{1}{2}\omega_{S_{1}}{\langle x-L_{1}\rangle }^{2}-\frac{1}{2}\omega_{S_{2}}{\langle x-L_{1}\rangle }^{2}\\ & +\frac{1}{2}\omega_{S_{2}}{\langle x-L_{12}\rangle }^{2}-\frac{1}{2}\omega_{S_{3}}{\langle x-L_{12}\rangle }^{2}+\frac{1}{2}\omega_{S_{3}}{\langle x-L_{123}\rangle }^{2}-\frac{1}{2}\omega_{S_{4}}{\langle x-L_{123}\rangle }^{2}\\ & +\frac{1}{2}\omega_{S_{4}}{\langle x-L_{1234}\rangle }^{2}-\frac{1}{2}\omega_{S_{5}}{\langle x-L_{1234}\rangle }^{2} \end{array}$$

To obtain the static deflections (*y_Sj_*) in each section of the nanogenerator, Equations (21)–(25) are substituted into Equations (6)–(10)and integrated using Macaulay’s function integration rules [52]. Next, the integration constants are obtained using the boundary conditions of Equations (11)–(15). Thus, the static deflection of the five sections of the nanogenerator:

For 0 < *x* < *L*_1_

(26) $$y_{S1}(x)\;=\;\frac{1}{{\left(EI_{z}\right)}_{S_{1}}}\left[-\frac{1}{2}M_{o}{\langle x-0\rangle }^{2}+\frac{1}{6}R_{1}{\langle x-0\rangle }^{3}-\frac{1}{24}\omega_{S_{1}}{\langle x-0\rangle }^{4}\right]$$

For *L*_1_ *< x < L*_12_

(27) $$\begin{array}{ll} y_{S_{2}}(x)\;= & \frac{1}{{\left(EI_{z}\right)}_{S_{2}}}[-\frac{1}{2}M_{o}{\langle x-0\rangle }^{2}+\frac{1}{6}R_{0}{\langle x-0\rangle }^{3}-\frac{1}{24}\omega_{S_{1}}{\langle x-0\rangle }^{4}+\frac{1}{24}\omega_{S_{1}}{\langle x-L_{1}\rangle }^{4}\\ & -\frac{1}{24}\omega_{S_{2}}{\langle x-L_{1}\rangle }^{4}]+C_{3}x+C_{4} \end{array}$$

Integration constants *C*_3_ and *C*_4_ are shown in Appendix A.

For *L*_12_ < *x* < *L*_123_

(28) $$\begin{array}{ll} y_{S_{3}}(x)= & \frac{1}{{\left(EI_{z}\right)}_{S_{3}}}[-\frac{1}{2}M_{o}{\langle x-0\rangle }^{2}+\frac{1}{6}R_{0}{\langle x-0\rangle }^{3}-\frac{1}{24}\omega_{S_{1}}{\langle x-0\rangle }^{4}+\frac{1}{24}\omega_{S_{1}}{\langle x-L_{1}\rangle }^{4}\\ & -\frac{1}{24}\omega_{S_{2}}{\langle x-L_{1}\rangle }^{4}+\frac{1}{24}\omega_{S_{2}}{\langle x-L_{12}\rangle }^{4}-\frac{1}{24}\omega_{S_{3}}{\langle x-L_{12}\rangle }^{4}]+C_{5}x+C_{6} \end{array}$$

Integration constants *C*_5_ and *C*_6_ are shown in Appendix A.

For *L*_123_ < *x* < *L*_1234_

(29) $$\begin{array}{ll} y_{S_{4}}(x)= & \frac{1}{{\left(EI_{z}\right)}_{S_{4}}}[-\frac{1}{2}M_{o}{\langle x-0\rangle }^{2}+\frac{1}{6}R_{0}{\langle x-0\rangle }^{3}-\frac{1}{24}\omega_{S_{1}}{\langle x-0\rangle }^{4}+\frac{1}{24}\omega_{S_{1}}{\langle x-L_{1}\rangle }^{4}\\ & -\frac{1}{24}\omega_{S_{2}}{\langle x-L_{1}\rangle }^{4}+\frac{1}{24}\omega_{S_{2}}{\langle x-L_{12}\rangle }^{4}-\frac{1}{24}\omega_{S_{3}}{\langle x-L_{12}\rangle }^{4}+\frac{1}{24}\omega_{S_{3}}{\langle x-L_{123}\rangle }^{4}\\ & -\frac{1}{24}\omega_{S_{4}}{\langle x-L_{123}\rangle }^{4}]+C_{7}x+C_{8} \end{array}$$

Integration constants *C*_7_ and *C*_8_ are shown in Appendix A.

For *L*_1234_ < *x* < *L*_12345_

(30) $$\begin{array}{ll} y_{S_{5}}(x)= & \frac{1}{{\left(EI_{z}\right)}_{S_{5}}}[-\frac{1}{2}M_{o}{\langle x-0\rangle }^{2}+\frac{1}{6}R_{0}{\langle x-0\rangle }^{3}-\frac{1}{24}\omega_{S_{1}}{\langle x-0\rangle }^{4}+\frac{1}{24}\omega_{S_{1}}{\langle x-L_{1}\rangle }^{4}\\ & -\frac{1}{24}\omega_{S_{2}}{\langle x-L_{1}\rangle }^{4}+\frac{1}{24}\omega_{S_{2}}{\langle x-L_{12}\rangle }^{4}-\frac{1}{24}\omega_{S_{3}}{\langle x-L_{12}\rangle }^{4}+\frac{1}{24}\omega_{S_{3}}{\langle x-L_{123}\rangle }^{4}\\ & -\frac{1}{24}\omega_{S_{4}}{\langle x-L_{123}\rangle }^{4}+\frac{1}{24}\omega_{S_{4}}{\langle x-L_{1234}\rangle }^{4}-\frac{1}{24}\omega_{S_{5}}{\langle x-L_{12345}\rangle }^{4}]+C_{9}x+C_{10} \end{array}$$

Integration constants *C*_9_ and *C*_10_ are shown in Appendix A.

The bending moment on the left support (*M*_0_) of the nanogenerator is calculated using the following boundary condition of deflection *y*_5_(*x*) in the right clamped support:

(31) $$y_{5}\left(L_{12345}\right)=0$$

(32) $$\begin{array}{ll} M_{0}= & -\frac{1}{6}(7L_{2}^{3}{\left(EI_{z}\right)}_{s_{1}}{\left(EI_{z}\right)}_{s_{3}}\omega_{s_{2}}+L_{2}^{3}{\left(EI_{z}\right)}_{s_{1}}{\left(EI_{z}\right)}_{s_{3}}\omega_{s_{4}}+3L_{2}^{2}L_{3}{\left(EI_{z}\right)}_{s_{1}}{\left(EI_{z}\right)}_{s_{2}}\omega_{s_{2}}\\ & +6L_{2}^{2}L_{3}{\left(EI_{z}\right)}_{s_{1}}{\left(EI_{z}\right)}_{s_{3}}\omega_{s_{2}}+3L_{2}^{2}L_{3}{\left(EI_{z}\right)}_{s_{1}}{\left(EI_{z}\right)}_{s_{3}}\omega_{s_{3}}+12L_{2}^{2}{\left(EI_{z}\right)}_{s_{1}}{\left(EI_{z}\right)}_{s_{3}}L_{1}\omega_{s_{1}}\\ & +9L_{2}^{2}{\left(EI_{z}\right)}_{s_{2}}{\left(EI_{z}\right)}_{s_{3}}L_{1}\omega_{s_{2}}+3L_{2}^{2}{\left(EI_{z}\right)}_{s_{2}}{\left(EI_{z}\right)}_{s_{3}}L_{1}\omega_{s_{4}}+3L_{2}L_{3}^{2}{\left(EI_{z}\right)}_{s_{1}}{\left(EI_{z}\right)}_{s_{2}}\omega_{s_{2}}\\ & +3L_{2}L_{3}^{2}{\left(EI_{z}\right)}_{s_{1}}{\left(EI_{z}\right)}_{s_{3}}\omega_{s_{3}}+6L_{2}L_{3}{\left(EI_{z}\right)}_{s_{1}}{\left(EI_{z}\right)}_{s_{2}}L_{1}\omega_{s_{2}}+6L_{2}L_{3}{\left(EI_{z}\right)}_{s_{1}}{\left(EI_{z}\right)}_{s_{3}}L_{1}\omega_{s_{1}}\\ & +6L_{2}L_{3}{\left(EI_{z}\right)}_{s_{2}}{\left(EI_{z}\right)}_{s_{3}}L_{1}\omega_{s_{2}}+6L_{2}L_{3}{\left(EI_{z}\right)}_{s_{2}}{\left(EI_{z}\right)}_{s_{3}}L_{1}\omega_{s_{3}}+6L_{2}{\left(EI_{z}\right)}_{s_{1}}{\left(EI_{z}\right)}_{s_{3}}L_{1}^{2}\omega_{s_{1}}\\ & +12L_{2}{\left(EI_{z}\right)}_{s_{2}}{\left(EI_{z}\right)}_{s_{3}}L_{1}^{2}\omega_{s_{1}}+3L_{2}{\left(EI_{z}\right)}_{s_{2}}{\left(EI_{z}\right)}_{s_{3}}L_{1}^{2}\omega_{s_{2}}+3L_{2}{\left(EI_{z}\right)}_{s_{2}}{\left(EI_{z}\right)}_{s_{3}}L_{1}^{2}\omega_{s_{4}}\\ & +L_{3}^{3}{\left(EI_{z}\right)}_{s_{1}}{\left(EI_{z}\right)}_{s_{2}}\omega_{s_{3}}+3L_{3}^{2}{\left(EI_{z}\right)}_{s_{1}}{\left(EI_{z}\right)}_{s_{2}}L_{1}\omega_{s_{1}}+3L_{3}^{2}{\left(EI_{z}\right)}_{s_{2}}{\left(EI_{z}\right)}_{s_{3}}L_{1}\omega_{s_{3}}\\ & +3L_{3}{\left(EI_{z}\right)}_{s_{1}}{\left(EI_{z}\right)}_{s_{2}}L_{1}^{2}\omega_{s_{1}}+6L_{3}{\left(EI_{z}\right)}_{s_{2}}{\left(EI_{z}\right)}_{s_{3}}L_{1}^{2}\omega_{s_{1}}+3L_{3}{\left(EI_{z}\right)}_{s_{2}}{\left(EI_{z}\right)}_{s_{3}}L_{1}^{2}\omega_{s_{3}}\\ & +7{\left(EI_{z}\right)}_{s_{2}}{\left(EI_{z}\right)}_{s_{3}}L_{1}^{3}\omega_{s_{1}}+{\left(EI_{z}\right)}_{s_{2}}{\left(EI_{z}\right)}_{s_{3}}L_{1}^{3}\omega_{s_{5}}-12L_{2}^{2}{\left(EI_{z}\right)}_{s_{1}}{\left(EI_{z}\right)}_{s_{3}}R_{0}\\ & -6L_{2}L_{3}{\left(EI_{z}\right)}_{s_{1}}{\left(EI_{z}\right)}_{s_{2}}R_{0}-6L_{2}L_{3}{\left(EI_{z}\right)}_{s_{1}}{\left(EI_{z}\right)}_{s_{3}}R_{0}-12L_{2}{\left(EI_{z}\right)}_{s_{1}}{\left(EI_{z}\right)}_{s_{3}}L_{1}R_{0}\\ & -12L_{2}{\left(EI_{z}\right)}_{s_{2}}{\left(EI_{z}\right)}_{s_{3}}L_{1}R_{0}-3L_{3}^{2}{\left(EI_{z}\right)}_{s_{1}}{\left(EI_{z}\right)}_{s_{2}}R_{0}-6L_{3}{\left(EI_{z}\right)}_{s_{1}}{\left(EI_{z}\right)}_{s_{2}}L_{1}R_{0}\\ & -6L_{3}{\left(EI_{z}\right)}_{s_{2}}{\left(EI_{z}\right)}_{s_{3}}L_{1}R_{0}-12{\left(EI_{z}\right)}_{s_{2}}{\left(EI_{z}\right)}_{s_{3}}L_{1}^{2}R_{0})/(2L_{2}{\left(EI_{z}\right)}_{s_{1}}{\left(EI_{z}\right)}_{s_{3}}\\ & +L_{3}{\left(EI_{z}\right)}_{s_{1}}{\left(EI_{z}\right)}_{s_{2}}+2{\left(EI_{z}\right)}_{s_{2}}{\left(EI_{z}\right)}_{s_{3}}L_{1}) \end{array}$$

The first bending resonant frequency of the nanogenerator is obtained by substituting Equations (3) and (4) into Equation (5). Table 1 depicts the geometric parameters of the different layers of the nanogenerator used in the analytical model. In addition, the moments and reactions of the two clamped supports, weight per unit length and bending stiffness for each section are indicated in Table 2. Considering the values of these parameters shown in Table 1 and Table 2, we determined the first bending frequency of the nanogenerator to be 110.94 Hz.

### 2.2. Finite Element Method (FEM) Models

**Figure 6:**
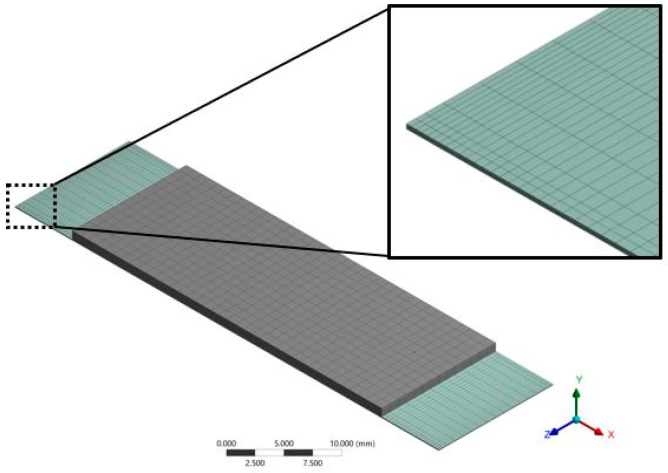
View of the mesh used in the finite element method (FEM) models of the piezoelectric nanogenerator.

FEM models are developed to predict the first resonant frequencies and vibration modes (modal analysis) of the nanogenerator. The deflection and mechanical stress of the nanogenerator are obtained through static structural analysis with the FEM models. Finally, the dynamic deflection, normal stress and output power are estimated by means of a harmonic response analysis. Figure 6 shows the mesh of the FEM model of the nanogenerator. Table 3 and Table 4 indicate the mechanical properties of the materials of the nanogenerator used in the analytical and FEM models. The mesh of the nanogenerator is performed with 20-node hexahedral SOLID186 elements.

**Figure 7:**
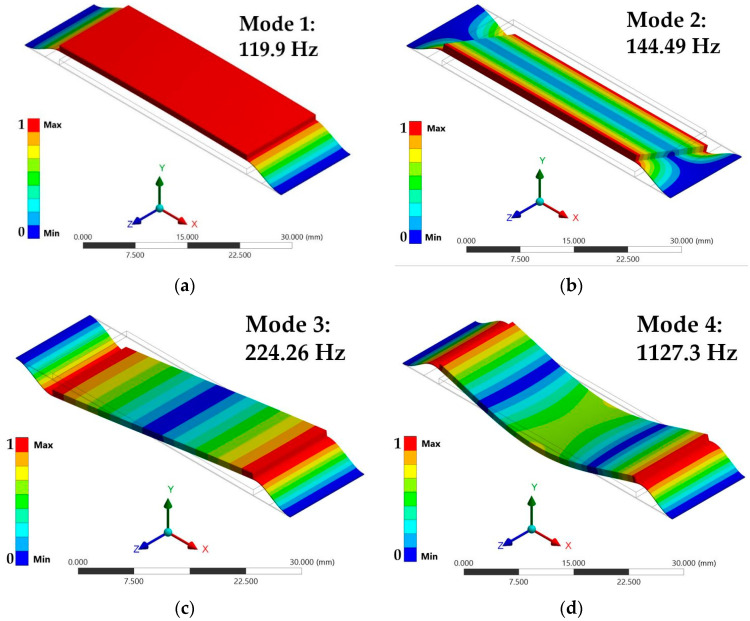
(**a**) First, (**b**) second, (**c**) third and (**d**) fourth vibration mode of the FEM models of the piezoelectric nanogenerator.

In the modal analysis, the first four vibration modes and resonant frequencies of the nanogenerator are obtained. Figure 7a depicts the first bending vibration mode of the nanogenerator, which occurs at 119.9 Hz. This value has a relative difference of −8.07% with respect to that of the analytical model. The second vibration mode has a torsional phase and a resonant frequency of 144.49 Hz (Figure 7b). The third (Figure 7c) and fourth (Figure 7d) vibration modes of the nanogenerator have resonant frequencies of 224.26 Hz and 1127.3 Hz, respectively. In Figure 7a–d, the scale bar indicates the normalized displacements of the nanogenerator.

The piezoelectric nanogenerator is designed to operate at atmospheric pressure and its damping ratio is given by:

(33) $$\zeta =\frac{1}{2Q_{a}}$$

where *Q_a_* is the quality factor of the nanogenerator and it can be estimated using [53,54,55]:

(34) $$Q_{a}=\frac{2}{3}\frac{\rho hb}{3\left[\mu_{0}/f_{r}+\left(b/2\right)\sqrt{\pi \rho_{0}\left(\mu_{0}/f_{r}\right)}\right]}$$

where *b*, *h*, and *ρ* are the width, thickness, and density of the double-clamped beam, respectively, *µ*_0_ and *ρ*0 are the viscosity and density of air, respectively, and *f_r_* is the resonant frequency of the double-clamped beam.

**Figure 8:**
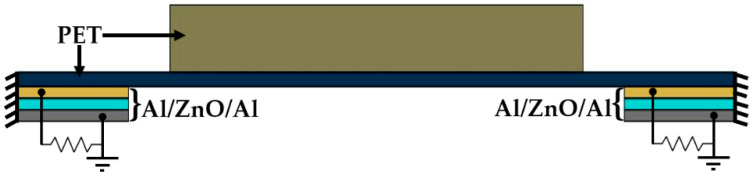
Schematic view of the electrical connection of the load resistance between the upper and lower electrodes of the piezoelectric nanogenerator.

Based on Equation (33), the quality factor and damping ratio of the nanogenerator have values of 99.98 and 5 × 10^−3^, respectively. On the other hand, a load resistance must be considered in the harmonic response analysis of the nanogenerator. This resistance is created through the CIRCU94 element available in the ANSYS software. The load resistor is connected between the upper and lower aluminum electrodes, as shown in Figure 8. The optimum load resistance (*R_opt_* = 32,458 Ω) is calculated by [56]:

(35) $$R_{opt}=\frac{1}{2\pi f_{r}C_{p}}$$

where *C_p_* is the capacitance of the ZnO layer.

(36) $$C_{p}=\frac{\varepsilon_{0}\varepsilon_{33}bL}{h}$$

The optimal resistance is required to determine the maximum power generated by the nanogenerator. This optimal resistance is defined as [56]:

(37) $$P=\frac{V^{2}}{R_{opt}}$$

where *V* is the generate voltage.

## 3. Results and Discussion

This section presents the results and discussion of the electromechanical performance of the piezoelectric nanogenerator determined by the analytical and FEM models.

**Figure 9:**
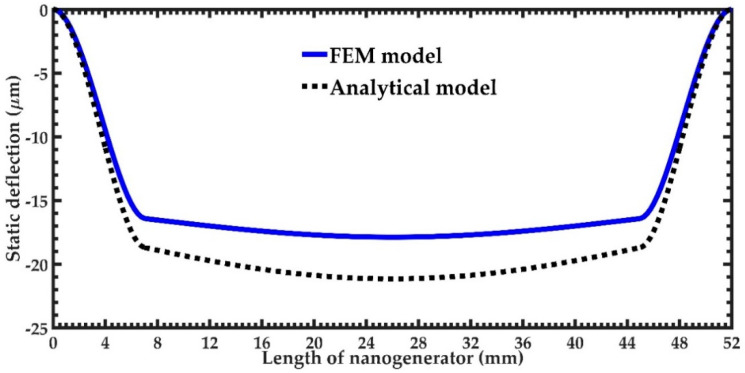
Static deflection of the piezoelectric nanogenerator obtained using the analytical and FEM models.

First, we determined the maximum deflections of the nanogenerator due to the static loads. An initial deflection of the nanogenerator equal to zero is assumed. For this, both analytical and FEM models considered the acceleration of Earth’s gravity along the *y*-axis. Figure 9 illustrates these static deflections of the nanogenerator estimated by the analytical and FEM models. The deflections calculated by the analytical model have similar responses to those of the FEM models.

**Figure 10:**
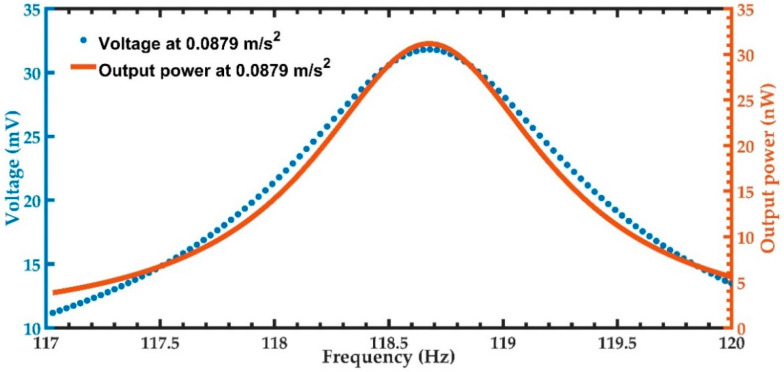
Voltage and output power generated by the nanogenerator considering vibrations (0.0879 m/s^2^) along the *y*-axis at office desk (0.0879 m/s^2^).

**Figure 11:**
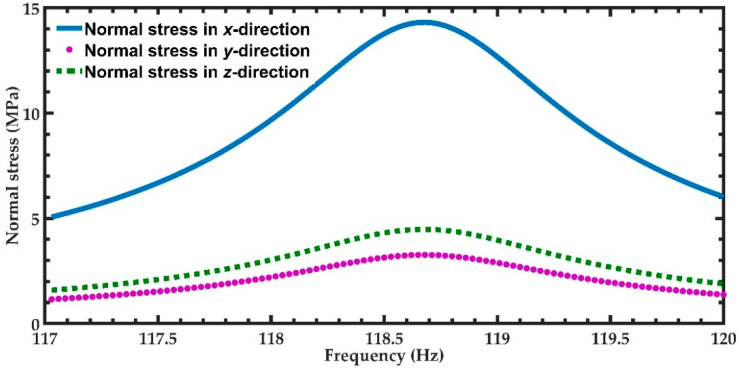
Maximum normal stresses of the nanogenerator considering an acceleration in *y*-direction of 0.0879 m/s^2^ and a frequency range from 117 to 120 Hz.

Next, a harmonic response analysis of the FEM model of the nanogenerator is studied considering an acceleration of vibration along the *y*-axis on the office desk of 0.0879 m/s^2^ [41]. Figure 10 depicts the voltage and output power generated through the load resistance of the nanogenerator. This voltage increases to a maximum value of 31.81 mV at the resonant frequency of 119.88 Hz. The maximum output power (31.18 nW) is generated by the piezoelectric layer located close to the left support of the nanogenerator. In addition, the two piezoelectric layers located on the two ends of the nanogenerator can generate a maximum current of 1.96 μA, total output power of 62.36 nW and output power density of 101.82 × 10^−3^ W/m^3^. Figure 11 shows the normal stresses along the axes *x*, *y* and *z* of the nanogenerator. The normal stress in the *x*-direction registers the maximum value of 14.31 MPa when the nanogenerator operates at resonance. This normal stress does not exceed the yield stress of ZnO (412 MPa) and PET (54.5 MPa) [57,58].

**Figure 12:**
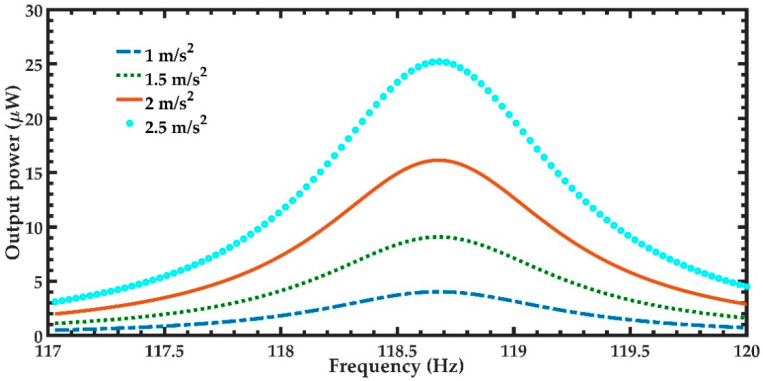
Maximum output power of the nanogenerator regarding four different accelerations in *y*-direction and a frequency range from 117 to 120 Hz.

**Figure 13:**
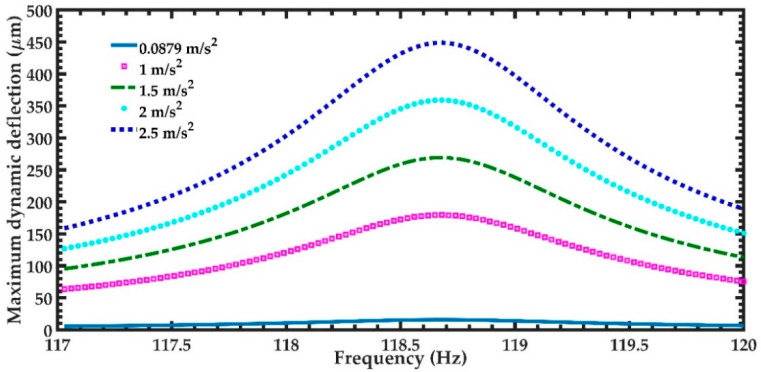
Maximum dynamic deflection of the nanogenerator considering four different accelerations in *y*-direction and a frequency range from 117 to 120 Hz.

**Figure 14:**
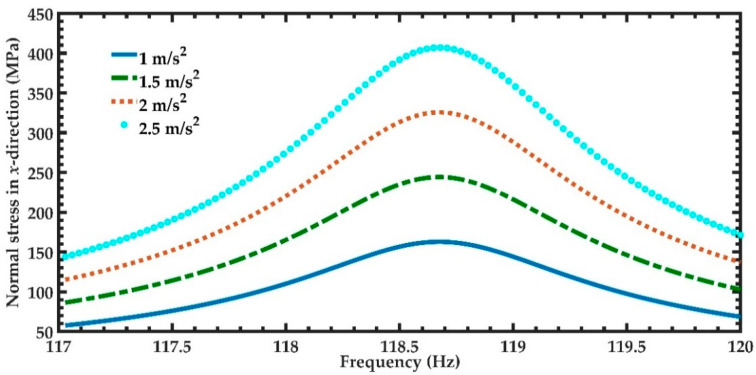
Maximum normal stress in *x*-direction of the nanogenerator regarding four different accelerations in *y*-direction and a frequency range from 117 to 120 Hz.

**Figure 15:**
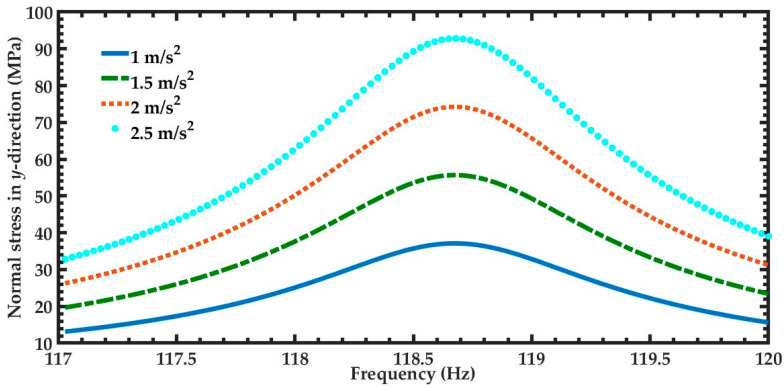
Maximum normal stress in *y*-direction of the nanogenerator considering four different accelerations in *y*-direction and a frequency range from 117 to 120 Hz.

**Figure 16:**
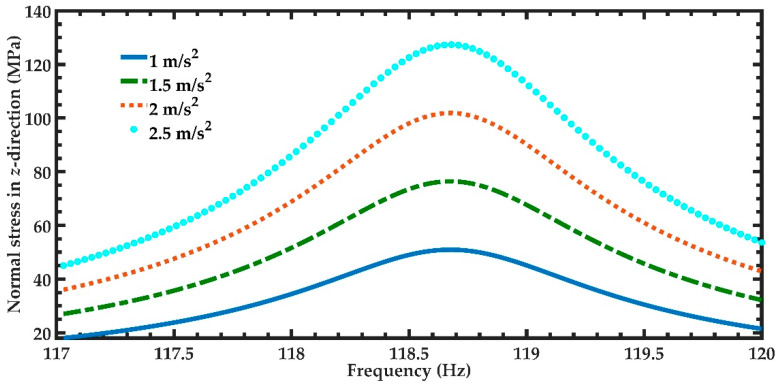
Maximum normal stress in *z*-direction of the nanogenerator regarding four different accelerations in *y*-direction and a frequency range from 117 to 120 Hz.

**Figure 17:**
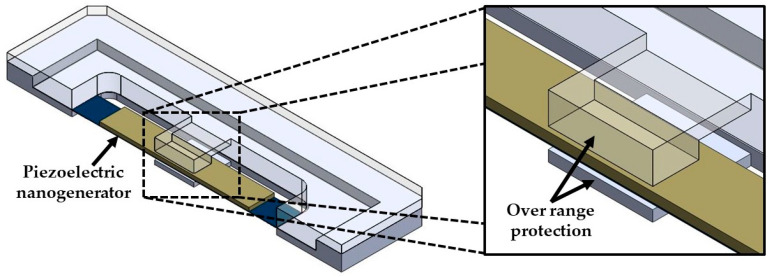
Cross-section view of the over range protection of the maximum deflection of the piezoelectric nanogenerator.

In order to predict the output power of the nanogenerator under different acceleration amplitudes in *y*-directions, four magnitudes of acceleration (1 m/s^2^, 1.5 m/s^2^, 2 m/s^2^, and 2.5 m/s^2^) along *y-*axis are considered (Figure 12). Figure 12 illustrates the power generated by the piezoelectric layer of the left end of the nanogenerator considering four different acceleration values. For the accelerations of 1 m/s^2^ and 2.5 m/s^2^, the output power of the nanogenerator at resonance can achieve values of 4.03 μW and 25.22 μW, respectively. Table 5 indicates the total output power and power density of the nanogenerator considering its two ZnO layers under different accelerations. For applications under accelerations in *y*-direction of 2.50 m/s^2^, the maximum output power and output power density of the nanogenerator at resonance under acceleration of 2.5 m/s^2^ are 50.44 μW and 82.36 W/m^3^, respectively. On the other hand, for applications with low accelerations in *y*-direction, the performance of nanogenerator decreases. In addition, the maximum dynamic deflection and the normal stresses in *x*, *y* and *z* directions of the nanogenerator are determined using a harmonic response analysis. Figure 13 depicts the maximum dynamic deflections of the nanogenerator for each acceleration value. The maximum dynamic deflection of the nanogenerator is close to 448 μm for an acceleration in *y*-direction of 2.5 m/s^2^. For this acceleration, the maximum normal stresses of the ZnO, PET, and aluminum layers are 407.09 MPa, 10.47 MPa and 204.23 MPa, respectively. These values do not overcome the yield stress of the ZnO (412 MPa), PET (54.5 MPa) and aluminum (280 MPa) layers. The maximum normal stresses occur on the ZnO layer close to the ends of the double-clamped beam. Figure 14, Figure 15 and Figure 16 show the maximum normal stresses in *x, y* and *z*-directions of the ZnO layer. However, the maximum normal stress in *x*-direction of the ZnO layer is close to its yield stress. To avoid the increase in this maximum normal stress, we propone an over range protection of the maximum deflection of the nanogenerator (see Figure 17). This over range protection is formed by two rigid structures that will limit the maximum deflection in *y*-direction of the nanogenerator to 448 μm. These two rigid structures are composed by two double-clamped beams, in which the upper beam will have a gap of 448 μm with respect to the outer surface of the seismic mass. On the other hand, the lower beam will have a gap of 448 μm with respect to the bottom surface of the PET. Thus, the maximum deflection of the nanogenerator is limited to 448 μm, although the accelerations in *y*-direction overcome 2.5 m/s^2^. This can constrain the maximum normal stress of the ZnO layer of the nanogenerator, keeping its operation safe.

The nanogenerator can use diode-bridge rectifiers to transform the alternating current to direct current. Thus, one array of the designed nanogenerators can include diode-bridge rectifiers to charge capacitors to DC voltage. Finally, this electrical charge could be applied to low-power consumption electronic devices. Moreover, the designers can enhance the nanogenerator behavior for different applications through the modification of its geometrical dimensions and materials. Additionally, the output voltages and power of nanogenerators can be increased when they operate at resonance with similar frequencies to those of ambient vibrations or mechanical motions. Therefore, the magnitudes of main design parameters of the nanogenerators can be adjusted by employing electromechanical models to increase their output voltage and power.

## 4. Conclusions

Analytical models to predict the first bending resonant frequency and deflections of a piezoelectric nanogenerator with multilayered cross-sections were presented. This nanogenerator was formed by a double-clamped beam composed of five sections with different layers. The nanogenerator was designed with a flexible PET substrate, ZnO layer, aluminum electrodes, and a seismic mass. The mechanical behavior of the nanogenerator was estimated using analytical models based on the Rayleigh method, the Euler–Bernoulli beam theory and the Macaulay method. Additionally, FEM models of the nanogenerator were used to predict its electromechanical behavior. The results of the mechanical behavior of the nanogenerator determined by the analytical models agreed well with respect to those of the FEM models. The first bending resonant frequency of the nanogenerator calculated with the analytical model had a relative difference of −8.07% in comparison to the FEM model. The nanogenerator can be used to convert the ambient mechanical vibrations under different accelerations into electrical energy. This electrical energy can be used to supply low-power consumption electronic devices. In addition, several nanogenerators can be used with diode-bridge rectifiers to charge capacitors to DC voltage. Furthermore, an over range protection of the maximum deflection of the nanogenerator was proposed to keep its safe operation during high accelerations.

Future research work will include the fabrication and characterization of piezoelectric nanogenerators and their implementation in potential applications.

## Figures and Tables

**Table 1:** Geometric parameters of the different layers of the piezoelectric nanogenerator.

Geometric Parameter	Magnitude
*L*_1_ = *L*_5_	4 mm
*L*_2_= *L*_4_	3 mm
*L*_3_	38 mm
*t*_1*S*1_ = *t*_2*S*1_ = *t*_3*S*1_ = *t*_1*S*5_ = *t*_2*S*5_ = *t*_3*S*5_	100 nm
*t*_4*S*1_ = *t*_4*S*2_ = *t*_4*S*3_ = *t*_4*S*4_ = *t*_4*S*5_	125 μm
*t*_5*S*3_	910 μm
*h*_1*S*1_ = *h*_1*S*5_	100 nm
*h*_2*S*1_ = *h*_2*S*5_	200 nm
*h*_3*S*1_ = *h*_3*S*5_	300 nm
*h*_4*S*1_ = *h*_4*S*2_ = *h*_4*S*3_ = *h*_4*S*4_ = *h*_4*S*5_	125.3 μm
*h*_5*S*3_	1035.3 μm

**Table 2:** Values for the bending moments, support reactions, weight per unit length and bending stiffness for each section of the piezoelectric nanogenerator.

Parameter	Magnitude
*M*_0_ = *M*_1_	14.5893 × 10^−6^ Nm
*R*_0_ = *R*_1_	3.9499 × 10^−3^ N
*ω_S_*_1_ = *ω_S_*_5_	24.1883 × 10^−3^ N/m
*ω_S_*_2_ = *ω_S_*_4_	24.0345 × 10^−3^ N/m
*ω_S_*_3_	199.0056 N/m
(*EI_z_*)*_S_*_1_ *=* (*EI_z_*)*_S_*_5_	6.8714 × 10^−6^ Nm^2^
(*EI_z_*)*_S_*_2_ = (*EI_z_*)*_S_*_4_	5.4687 × 10^−6^ Nm^2^
(*EI_z_*)*_S_*_3_	3.1044 × 10^−3^ Nm^2^

**Table 3:** Properties of the piezoelectric nanogenerator used in the analytical and finite element method (FEM) models [44].

Material Property	PET	Aluminum	ZnO
Young modulus *E* (GPa)	2.4	71	137
Density *ρ* (k/m^3^)	1400	2770	5665
Poisson ratio *υ*	0.36	0.33	0.34

**Table 4:** Zinc oxide (ZnO) piezoelectric matrices used in the FEM models of the piezoelectric nanogenerator [44].

ZnO piezoelectric stress matrix (e)
$\left[e\right]={\left[\begin{array}{ccc} 0 & 0 & -0.570878\\ 0 & 0 & -0.570878\\ 0 & 0 & 0.428446\\ 0 & 0 & 0\\ 0 & -0.480816 & 0\\ -0.480816 & 0 & 0 \end{array}\right]}_{\;6\times 3}\frac{C}{m^{2}}$
ZnO piezoelectric dielectric matrix (*ε_r_*) under the constant strain.
$\left[\varepsilon_{r}\right]={\left[\begin{array}{ccc} 7.57 & 0 & 0\\ 0 & 7.57 & 0\\ 0 & 0 & 8.31 \end{array}\right]}_{\;3\times 3}$

**Table 5:** Total output power and power density of the nanogenerator under different accelerations in *y*-direction.

Acceleration (m/s^2^)	Total Output Power (µW)	Total Output Power Density (W/m^3^)
8.79 × 10^−2^	62.36 × 10^−3^	101.82 × 10^−3^
1.0	8.06	13.16
1.5	18.14	29.62
2.0	32.28	52.71
2.5	50.44	82.36

## Appendix A

This appendix shows the integration constants (*C*_3_, *C*_4_, *C*_5_, *C*_6_, *C*_7_, *C*_8_, *C*_9_, and *C*_10_) of the static deflections of the piezoelectric nanogenerator, which were determined by proposed analytical modeling.

Integration constants *C*_3_ and *C*_4_ of the static deflection (*y_S_*_2_) of the nanogenerator:

(A1) $$\begin{array}{ll} C_{3}= & \frac{1}{6}\frac{1}{{\left(EI_{z}\right)}_{s_{1}}{\left(EI_{z}\right)}_{s_{2}}}(L_{s_{1}}({\left(EI_{z}\right)}_{s_{1}}L_{1}^{2}\omega_{s_{1}}-{\left(EI_{z}\right)}_{s_{2}}L_{1}^{2}\omega_{s_{1}}-3{\left(EI_{z}\right)}_{s_{1}}L_{1}R_{0}\\ & +3{\left(EI_{z}\right)}_{s_{2}}L_{1}R_{0}+6{\left(EI_{z}\right)}_{s_{1}}M_{0}-6{\left(EI_{z}\right)}_{s_{2}}M_{0})) \end{array}$$

(A2) $$\begin{array}{ll} C_{4}= & -\frac{1}{24}\frac{1}{{\left(EI_{z}\right)}_{s_{1}}{\left(EI_{z}\right)}_{s_{2}}}(L_{1}^{2}(3{\left(EI_{z}\right)}_{s_{1}}L_{1}^{2}\omega_{s_{1}}-3{\left(EI_{z}\right)}_{s_{2}}L_{1}^{2}\omega_{s_{1}}-8{\left(EI_{z}\right)}_{s_{1}}L_{1}R_{0}\\ & +8{\left(EI_{z}\right)}_{s_{2}}L_{1}R_{0}+12{\left(EI_{z}\right)}_{s_{1}}M_{0}-12{\left(EI_{z}\right)}_{s_{2}}M_{0})) \end{array}$$

Integration constants *C*_5_ and *C*_6_ of the static deflection (*y_S_*_3_) of the nanogenerator:

(A3) $$\begin{array}{ll} C_{5}= & \frac{1}{6}\frac{1}{{\left(EI_{z}\right)}_{s_{1}}{\left(EI_{z}\right)}_{s_{2}}{\left(EI_{z}\right)}_{s_{3}}}(L_{2}^{3}{\left(EI_{z}\right)}_{s_{1}}{\left(EI_{z}\right)}_{s_{2}}\omega_{S_{2}}-L_{2}^{3}{\left(EI_{z}\right)}_{s_{1}}{\left(EI_{z}\right)}_{s_{3}}\omega_{S_{2}}\\ & +3L_{2}^{2}{\left(EI_{z}\right)}_{s_{1}}{\left(EI_{z}\right)}_{s_{2}}L_{1}\omega_{S_{1}}-3L_{2}^{2}{\left(EI_{z}\right)}_{s_{1}}{\left(EI_{z}\right)}_{s_{3}}L_{1}\omega_{S_{1}}+3L_{2}{\left(EI_{z}\right)}_{s_{1}}{\left(EI_{z}\right)}_{s_{2}}L_{1}^{2}\omega_{S_{1}}\\ & +3L_{2}^{2}{\left(EI_{z}\right)}_{s_{1}}{\left(EI_{z}\right)}_{s_{2}}L_{1}\omega_{S_{1}}-3L_{2}^{2}{\left(EI_{z}\right)}_{s_{1}}{\left(EI_{z}\right)}_{s_{3}}L_{1}\omega_{S_{1}}+3L_{2}{\left(EI_{z}\right)}_{s_{1}}{\left(EI_{z}\right)}_{s_{2}}L_{1}^{2}\omega_{S_{1}}\\ & +3L_{2}^{2}{\left(EI_{z}\right)}_{s_{1}}{\left(EI_{z}\right)}_{s_{2}}L_{1}\omega_{S_{1}}-3L_{2}^{2}{\left(EI_{z}\right)}_{s_{1}}{\left(EI_{z}\right)}_{s_{3}}L_{1}\omega_{S_{1}}+3L_{2}{\left(EI_{z}\right)}_{s_{1}}{\left(EI_{z}\right)}_{s_{2}}L_{1}^{2}\omega_{S_{1}}\\ & -3L_{2}{\left(EI_{z}\right)}_{s_{1}}{\left(EI_{z}\right)}_{s_{3}}L_{{}_{1}}^{2}\omega_{S_{1}}+{\left(EI_{z}\right)}_{s_{1}}{\left(EI_{z}\right)}_{s_{2}}L_{{}_{1}}^{3}\omega_{S_{1}}-{\left(EI_{z}\right)}_{s_{2}}{\left(EI_{z}\right)}_{s_{3}}L_{{}_{1}}^{3}\omega_{S_{1}}\\ & -3L_{{}_{2}}^{2}{\left(EI_{z}\right)}_{s_{1}}{\left(EI_{z}\right)}_{s_{2}}R_{0}+3L_{{}_{2}}^{2}{\left(EI_{z}\right)}_{s_{1}}{\left(EI_{z}\right)}_{s_{3}}R_{0}-6L_{2}{\left(EI_{z}\right)}_{s_{1}}{\left(EI_{z}\right)}_{s_{2}}L_{1}R_{0}\\ & +6L_{2}{\left(EI_{z}\right)}_{s_{1}}{\left(EI_{z}\right)}_{s_{3}}L_{1}R_{0}-3{\left(EI_{z}\right)}_{s_{1}}{\left(EI_{z}\right)}_{s_{2}}L_{{}_{1}}^{2}R_{0}+3{\left(EI_{z}\right)}_{s_{2}}{\left(EI_{z}\right)}_{s_{3}}L_{{}_{1}}^{2}R_{0}\\ & +6L_{2}{\left(EI_{z}\right)}_{s_{1}}{\left(EI_{z}\right)}_{s_{2}}L_{{}_{1}}^{2}M_{0}-6L_{2}{\left(EI_{z}\right)}_{s_{1}}{\left(EI_{z}\right)}_{s_{3}}L_{{}_{1}}^{2}M_{0}+6{\left(EI_{z}\right)}_{s_{1}}{\left(EI_{z}\right)}_{s_{2}}L_{1}M_{0}\\ & -6{\left(EI_{z}\right)}_{s_{2}}{\left(EI_{z}\right)}_{s_{3}}L_{1}M_{0}) \end{array}$$

(A4) $$\begin{array}{ll} C_{6}= & -\frac{1}{24}\frac{1}{{\left(EI_{z}\right)}_{s_{1}}{\left(EI_{z}\right)}_{s_{2}}{\left(EI_{z}\right)}_{s_{3}}}(3L_{2}^{4}{\left(EI_{z}\right)}_{s_{1}}{\left(EI_{z}\right)}_{s_{2}}\omega_{S_{2}}-3L_{2}^{4}{\left(EI_{z}\right)}_{s_{1}}{\left(EI_{z}\right)}_{s_{3}}\omega_{S_{2}}\\ & +8L_{2}^{3}{\left(EI_{z}\right)}_{s_{1}}{\left(EI_{z}\right)}_{s_{2}}L_{1}\omega_{S_{1}}+4L_{2}^{3}{\left(EI_{z}\right)}_{s_{1}}{\left(EI_{z}\right)}_{s_{2}}L_{1}\omega_{S_{2}}-8L_{2}^{3}{\left(EI_{z}\right)}_{s_{1}}{\left(EI_{z}\right)}_{s_{3}}L_{1}\omega_{S_{1}}\\ & -4L_{2}^{3}{\left(EI_{z}\right)}_{s_{1}}{\left(EI_{z}\right)}_{s_{3}}L_{1}\omega_{S_{2}}+18L_{2}^{2}{\left(EI_{z}\right)}_{s_{1}}{\left(EI_{z}\right)}_{s_{2}}L_{1}^{2}\omega_{S_{1}}-18L_{2}^{2}{\left(EI_{z}\right)}_{s_{1}}{\left(EI_{z}\right)}_{s_{3}}L_{1}^{2}\omega_{S_{1}}\\ & +12L_{2}{\left(EI_{z}\right)}_{s_{1}}{\left(EI_{z}\right)}_{s_{2}}L_{1}^{3}\omega_{S_{1}}-12L_{2}{\left(EI_{z}\right)}_{s_{1}}{\left(EI_{z}\right)}_{s_{3}}L_{1}^{3}\omega_{S_{1}}+3{\left(EI_{z}\right)}_{s_{1}}{\left(EI_{z}\right)}_{s_{2}}L_{1}^{4}\omega_{S_{1}}\\ & -3{\left(EI_{z}\right)}_{s_{2}}{\left(EI_{z}\right)}_{s_{3}}L_{1}^{4}\omega_{S_{1}}-8L_{2}^{3}{\left(EI_{z}\right)}_{s_{1}}{\left(EI_{z}\right)}_{s_{2}}R_{0}+8L_{2}^{3}{\left(EI_{z}\right)}_{s_{1}}{\left(EI_{z}\right)}_{s_{3}}R_{0}\\ & -24L_{2}^{2}{\left(EI_{z}\right)}_{s_{1}}{\left(EI_{z}\right)}_{s_{2}}L_{1}R_{0}+24L_{2}^{2}{\left(EI_{z}\right)}_{s_{1}}{\left(EI_{z}\right)}_{s_{3}}L_{1}R_{0}-24L_{2}{\left(EI_{z}\right)}_{s_{1}}{\left(EI_{z}\right)}_{s_{2}}L_{1}^{2}R_{0}\\ & +24L_{2}{\left(EI_{z}\right)}_{s_{1}}{\left(EI_{z}\right)}_{s_{3}}L_{1}^{2}R_{0}-8{\left(EI_{z}\right)}_{s_{1}}{\left(EI_{z}\right)}_{s_{2}}L_{1}^{3}R_{0}+8{\left(EI_{z}\right)}_{s_{2}}{\left(EI_{z}\right)}_{s_{3}}L_{1}^{3}R_{0}\\ & +12L_{2}^{2}{\left(EI_{z}\right)}_{s_{1}}{\left(EI_{z}\right)}_{s_{2}}M_{0}-12L_{2}^{2}{\left(EI_{z}\right)}_{s_{1}}{\left(EI_{z}\right)}_{s_{3}}M_{0}+24L_{2}{\left(EI_{z}\right)}_{s_{1}}{\left(EI_{z}\right)}_{s_{2}}L_{1}M_{0}\\ & -24L_{2}{\left(EI_{z}\right)}_{s_{1}}{\left(EI_{z}\right)}_{s_{3}}L_{1}M_{0}+12{\left(EI_{z}\right)}_{s_{1}}{\left(EI_{z}\right)}_{s_{2}}L_{1}^{2}M_{0}-12{\left(EI_{z}\right)}_{s_{2}}{\left(EI_{z}\right)}_{s_{3}}L_{1}^{2}M_{0}) \end{array}$$

Integration constants *C*_7_ and *C*_8_ of the static deflection (*y_S_*_4_) of the nanogenerator:

(A5) $$\begin{array}{ll} C_{7}= & -\frac{1}{6}\frac{1}{{\left(EI_{z}\right)}_{s_{1}}{\left(EI_{z}\right)}_{s_{2}}{\left(EI_{z}\right)}_{s_{3}}}(3L_{2}^{2}L_{3}{\left(EI_{z}\right)}_{s_{1}}{\left(EI_{z}\right)}_{s_{2}}\omega_{S_{2}}-3L_{2}^{2}L_{3}{\left(EI_{z}\right)}_{s_{1}}{\left(EI_{z}\right)}_{s_{3}}\omega_{S_{2}}\\ & \;+3L_{2}L_{3}^{2}{\left(EI_{z}\right)}_{s_{1}}{\left(EI_{z}\right)}_{s_{2}}\omega_{S_{2}}-3L_{2}L_{3}^{2}{\left(EI_{z}\right)}_{s_{1}}{\left(EI_{z}\right)}_{s_{3}}\omega_{S_{2}}+6L_{2}L_{3}{\left(EI_{z}\right)}_{s_{1}}{\left(EI_{z}\right)}_{s_{2}}L_{1}\omega_{S_{1}}\\ & -6L_{2}L_{3}{\left(EI_{z}\right)}_{s_{1}}{\left(EI_{z}\right)}_{s_{3}}L_{1}\omega_{S_{1}}+L_{3}^{3}{\left(EI_{z}\right)}_{s_{1}}{\left(EI_{z}\right)}_{s_{2}}\omega_{S_{3}}-L_{3}^{3}{\left(EI_{z}\right)}_{s_{1}}{\left(EI_{z}\right)}_{s_{3}}\omega_{S_{3}}\\ & +3L_{3}^{2}{\left(EI_{z}\right)}_{s_{1}}{\left(EI_{z}\right)}_{s_{2}}L_{1}\omega_{S_{1}}-3L_{3}^{2}{\left(EI_{z}\right)}_{s_{1}}{\left(EI_{z}\right)}_{s_{3}}L_{1}\omega_{S_{1}}+3L_{3}{\left(EI_{z}\right)}_{s_{1}}{\left(EI_{z}\right)}_{s_{2}}L_{1}^{2}\omega_{S_{1}}\\ & -3L_{3}{\left(EI_{z}\right)}_{s_{1}}{\left(EI_{z}\right)}_{s_{3}}L_{1}^{2}\omega_{S_{1}}-{\left(EI_{z}\right)}_{s_{1}}{\left(EI_{z}\right)}_{s_{3}}L_{1}^{3}\omega_{S_{1}}+{\left(EI_{z}\right)}_{s_{2}}{\left(EI_{z}\right)}_{s_{3}}L_{1}^{3}\omega_{S_{1}}\\ & -6L_{2}L_{3}{\left(EI_{z}\right)}_{s_{1}}{\left(EI_{z}\right)}_{s_{2}}R_{0}+6L_{2}L_{3}{\left(EI_{z}\right)}_{s_{1}}{\left(EI_{z}\right)}_{s_{3}}R_{0}-3L_{3}^{2}{\left(EI_{z}\right)}_{s_{1}}{\left(EI_{z}\right)}_{s_{2}}R_{0}\\ & +3L_{3}^{2}{\left(EI_{z}\right)}_{s_{1}}{\left(EI_{z}\right)}_{s_{3}}R_{0}-6L_{3}{\left(EI_{z}\right)}_{s_{1}}{\left(EI_{z}\right)}_{s_{2}}L_{1}R_{0}+6L_{3}{\left(EI_{z}\right)}_{s_{1}}{\left(EI_{z}\right)}_{s_{3}}L_{1}R_{0}\\ & +3{\left(EI_{z}\right)}_{s_{1}}{\left(EI_{z}\right)}_{s_{3}}L_{1}^{2}R_{0}-3{\left(EI_{z}\right)}_{s_{2}}{\left(EI_{z}\right)}_{s_{3}}L_{1}^{2}R_{0}+6L_{3}{\left(EI_{z}\right)}_{s_{1}}{\left(EI_{z}\right)}_{s_{2}}M_{0}\\ & -6L_{3}{\left(EI_{z}\right)}_{s_{1}}{\left(EI_{z}\right)}_{s_{3}}M_{0}-6{\left(EI_{z}\right)}_{s_{1}}{\left(EI_{z}\right)}_{s_{3}}L_{1}M_{0}+6{\left(EI_{z}\right)}_{s_{2}}{\left(EI_{z}\right)}_{s_{3}}L_{1}M_{0}) \end{array}$$

(A6) $$\begin{array}{ll} C_{8}= & \frac{1}{24}\frac{1}{{\left(EI_{z}\right)}_{s_{1}}{\left(EI_{z}\right)}_{s_{2}}{\left(EI_{z}\right)}_{s_{3}}}(12L_{2}^{3}L_{3}{\left(EI_{z}\right)}_{s_{1}}{\left(EI_{z}\right)}_{s_{2}}\omega_{S_{2}}-12L_{2}^{3}L_{3}{\left(EI_{z}\right)}_{s_{1}}{\left(EI_{z}\right)}_{s_{3}}\omega_{S_{2}}\\ & \;+18L_{2}^{2}L_{3}^{2}{\left(EI_{z}\right)}_{s_{1}}{\left(EI_{z}\right)}_{s_{2}}\omega_{S_{2}}-18L_{2}^{2}L_{3}^{2}{\left(EI_{z}\right)}_{s_{1}}{\left(EI_{z}\right)}_{s_{3}}\omega_{S_{2}}+24L_{2}^{2}L_{3}{\left(EI_{z}\right)}_{s_{1}}{\left(EI_{z}\right)}_{s_{2}}L_{1}\omega_{S_{1}}\\ & \;+12L_{2}^{2}L_{3}{\left(EI_{z}\right)}_{s_{1}}{\left(EI_{z}\right)}_{s_{2}}L_{1}\omega_{S_{2}}-24L_{2}^{2}L_{3}{\left(EI_{z}\right)}_{s_{1}}{\left(EI_{z}\right)}_{s_{3}}L_{1}\omega_{S_{1}}-12L_{2}^{2}L_{3}{\left(EI_{z}\right)}_{s_{1}}{\left(EI_{z}\right)}_{s_{3}}L_{1}\omega_{S_{2}}\\ & \;+8L_{2}L_{3}^{3}{\left(EI_{z}\right)}_{s_{1}}{\left(EI_{z}\right)}_{s_{2}}\omega_{S_{2}}+4L_{2}L_{3}^{3}{\left(EI_{z}\right)}_{s_{1}}{\left(EI_{z}\right)}_{s_{2}}\omega_{S_{3}}-8L_{2}L_{3}^{3}{\left(EI_{z}\right)}_{s_{1}}{\left(EI_{z}\right)}_{s_{3}}\omega_{S_{2}}\\ & -4L_{2}L_{3}^{3}{\left(EI_{z}\right)}_{s_{1}}{\left(EI_{z}\right)}_{s_{3}}\omega_{S_{3}}+24L_{2}L_{3}^{2}{\left(EI_{z}\right)}_{s_{1}}{\left(EI_{z}\right)}_{s_{2}}L_{1}\omega_{S_{1}}+12L_{2}L_{3}^{2}{\left(EI_{z}\right)}_{s_{1}}{\left(EI_{z}\right)}_{s_{2}}L_{1}\omega_{S_{2}}\\ & -24L_{2}L_{3}^{2}{\left(EI_{z}\right)}_{s_{1}}{\left(EI_{z}\right)}_{s_{3}}L_{1}\omega_{S_{1}}-12L_{2}L_{3}^{2}{\left(EI_{z}\right)}_{s_{1}}{\left(EI_{z}\right)}_{s_{3}}L_{1}\omega_{S_{2}}+36L_{2}L_{3}{\left(EI_{z}\right)}_{s_{1}}{\left(EI_{z}\right)}_{s_{2}}L_{1}^{2}\omega_{S_{1}}\\ & -36L_{2}L_{3}{\left(EI_{z}\right)}_{s_{1}}{\left(EI_{z}\right)}_{s_{3}}L_{1}^{2}\omega_{S_{1}}+3L_{3}^{4}{\left(EI_{z}\right)}_{s_{1}}{\left(EI_{z}\right)}_{s_{2}}\omega_{S_{3}}-3L_{3}^{4}{\left(EI_{z}\right)}_{s_{1}}{\left(EI_{z}\right)}_{s_{3}}\omega_{S_{3}}\\ & -4L_{3}^{3}{\left(EI_{z}\right)}_{s_{1}}{\left(EI_{z}\right)}_{s_{3}}L_{1}\omega_{S_{3}}+18L_{3}^{2}{\left(EI_{z}\right)}_{s_{1}}{\left(EI_{z}\right)}_{s_{2}}L_{1}^{2}\omega_{S_{1}}-18L_{3}^{2}{\left(EI_{z}\right)}_{s_{1}}{\left(EI_{z}\right)}_{s_{3}}L_{1}^{2}\omega_{S_{1}}\\ & +12L_{3}{\left(EI_{z}\right)}_{s_{1}}{\left(EI_{z}\right)}_{s_{2}}L_{1}^{3}\omega_{S_{1}}-12L_{3}{\left(EI_{z}\right)}_{s_{1}}{\left(EI_{z}\right)}_{s_{3}}L_{1}^{3}\omega_{S_{1}}-3{\left(EI_{z}\right)}_{s_{1}}{\left(EI_{z}\right)}_{s_{3}}L_{1}^{4}\omega_{S_{1}}\\ & +3{\left(EI_{z}\right)}_{s_{2}}{\left(EI_{z}\right)}_{s_{3}}L_{1}^{4}\omega_{S_{1}}-24L_{2}^{2}L_{3}{\left(EI_{z}\right)}_{s_{1}}{\left(EI_{z}\right)}_{s_{2}}R_{0}+24L_{2}^{2}L_{3}{\left(EI_{z}\right)}_{s_{1}}{\left(EI_{z}\right)}_{s_{3}}R_{0}\\ & -24L_{2}L_{3}^{2}{\left(EI_{z}\right)}_{s_{1}}{\left(EI_{z}\right)}_{s_{2}}R_{0}+24L_{2}L_{3}^{2}{\left(EI_{z}\right)}_{s_{1}}{\left(EI_{z}\right)}_{s_{3}}R_{0}-48L_{2}L_{3}{\left(EI_{z}\right)}_{s_{1}}{\left(EI_{z}\right)}_{s_{2}}L_{1}R_{0}\\ & +48L_{2}L_{3}{\left(EI_{z}\right)}_{s_{1}}{\left(EI_{z}\right)}_{s_{3}}L_{1}R_{0}-8L_{3}^{3}{\left(EI_{z}\right)}_{s_{1}}{\left(EI_{z}\right)}_{s_{2}}R_{0}+8L_{3}^{3}{\left(EI_{z}\right)}_{s_{1}}{\left(EI_{z}\right)}_{s_{3}}R_{0}\\ & -24L_{3}^{2}{\left(EI_{z}\right)}_{s_{1}}{\left(EI_{z}\right)}_{s_{2}}L_{1}R_{0}+24L_{3}^{2}{\left(EI_{z}\right)}_{s_{1}}{\left(EI_{z}\right)}_{s_{3}}L_{1}R_{0}-24L_{3}{\left(EI_{z}\right)}_{s_{1}}{\left(EI_{z}\right)}_{s_{2}}L_{1}^{2}R_{0}\\ & +24L_{3}{\left(EI_{z}\right)}_{s_{1}}{\left(EI_{z}\right)}_{s_{3}}L_{1}^{2}R_{0}+8{\left(EI_{z}\right)}_{s_{1}}{\left(EI_{z}\right)}_{s_{3}}L_{1}^{3}R_{0}-8{\left(EI_{z}\right)}_{s_{2}}{\left(EI_{z}\right)}_{s_{3}}L_{1}^{3}R_{0}\\ & +24L_{2}L_{3}{\left(EI_{z}\right)}_{s_{1}}{\left(EI_{z}\right)}_{s_{2}}M_{0}-24L_{2}L_{3}{\left(EI_{z}\right)}_{s_{1}}{\left(EI_{z}\right)}_{s_{3}}M_{0}+12L_{3}^{2}{\left(EI_{z}\right)}_{s_{1}}{\left(EI_{z}\right)}_{s_{2}}M_{0}\\ & -12L_{3}^{2}{\left(EI_{z}\right)}_{s_{1}}{\left(EI_{z}\right)}_{s_{3}}M_{0}+24L_{3}{\left(EI_{z}\right)}_{s_{1}}{\left(EI_{z}\right)}_{s_{2}}L_{1}M_{0}-24L_{3}{\left(EI_{z}\right)}_{s_{1}}{\left(EI_{z}\right)}_{s_{3}}L_{1}M_{0}\\ & -12{\left(EI_{z}\right)}_{s_{1}}{\left(EI_{z}\right)}_{s_{3}}L_{1}^{2}M_{0}+12{\left(EI_{z}\right)}_{s_{2}}{\left(EI_{z}\right)}_{s_{3}}L_{1}^{2}M_{0}) \end{array}$$

Integration constants *C*_9_ and *C*_10_ of the static deflection (*y_S_*_5_) of the nanogenerator.

(A7) $$\begin{array}{ll} C_{9}= & -\frac{1}{6}\frac{1}{{\left(EI_{z}\right)}_{s_{1}}{\left(EI_{z}\right)}_{s_{2}}{\left(EI_{z}\right)}_{s_{3}}}(7L_{2}^{3}{\left(EI_{z}\right)}_{s_{1}}{\left(EI_{z}\right)}_{s_{3}}\omega_{S_{2}}+L_{2}^{3}{\left(EI_{z}\right)}_{s_{1}}{\left(EI_{z}\right)}_{s_{3}}\omega_{S_{4}}\\ & -7L_{2}^{3}{\left(EI_{z}\right)}_{s_{2}}{\left(EI_{z}\right)}_{s_{3}}\omega_{S_{2}}-L_{2}^{3}{\left(EI_{z}\right)}_{s_{2}}{\left(EI_{z}\right)}_{s_{3}}\omega_{S_{4}}+3L_{2}^{2}L_{3}{\left(EI_{z}\right)}_{s_{1}}{\left(EI_{z}\right)}_{s_{2}}\omega_{S_{2}}\\ & +6L_{2}^{2}L_{3}{\left(EI_{z}\right)}_{s_{1}}{\left(EI_{z}\right)}_{s_{3}}\omega_{S_{2}}+3L_{2}^{2}L_{3}{\left(EI_{z}\right)}_{s_{1}}{\left(EI_{z}\right)}_{s_{3}}\omega_{S_{3}}-9L_{2}^{2}L_{3}{\left(EI_{z}\right)}_{s_{2}}{\left(EI_{z}\right)}_{s_{3}}\omega_{S_{2}}\\ & -3L_{2}^{2}L_{3}{\left(EI_{z}\right)}_{s_{2}}{\left(EI_{z}\right)}_{s_{3}}\omega_{S_{3}}+12L_{2}^{2}{\left(EI_{z}\right)}_{s_{1}}{\left(EI_{z}\right)}_{s_{3}}L_{1}\omega_{S_{1}}-12L_{2}^{2}{\left(EI_{z}\right)}_{s_{2}}{\left(EI_{z}\right)}_{s_{3}}L_{1}\omega_{S_{1}}\\ & +3L_{2}L_{3}^{2}{\left(EI_{z}\right)}_{s_{1}}{\left(EI_{z}\right)}_{s_{2}}\omega_{S_{2}}+3L_{2}L_{3}^{2}{\left(EI_{z}\right)}_{s_{1}}{\left(EI_{z}\right)}_{s_{3}}\omega_{S_{3}}-3L_{2}L_{3}^{2}{\left(EI_{z}\right)}_{s_{2}}{\left(EI_{z}\right)}_{s_{3}}\omega_{S_{2}}\\ & -3L_{2}L_{3}^{2}{\left(EI_{z}\right)}_{s_{2}}{\left(EI_{z}\right)}_{s_{3}}\omega_{S_{3}}+6L_{2}L_{3}{\left(EI_{z}\right)}_{s_{1}}{\left(EI_{z}\right)}_{s_{2}}L_{1}\omega_{S_{1}}+6L_{2}L_{3}{\left(EI_{z}\right)}_{s_{1}}{\left(EI_{z}\right)}_{s_{3}}L_{1}\omega_{S_{1}}\\ & -12L_{2}L_{3}{\left(EI_{z}\right)}_{s_{2}}{\left(EI_{z}\right)}_{s_{3}}L_{1}\omega_{S_{1}}+6L_{2}{\left(EI_{z}\right)}_{s_{1}}{\left(EI_{z}\right)}_{s_{3}}L_{1}^{2}\omega_{S_{1}}-6L_{2}{\left(EI_{z}\right)}_{s_{2}}{\left(EI_{z}\right)}_{s_{3}}L_{1}^{2}\omega_{S_{1}}\\ & +L_{3}^{3}{\left(EI_{z}\right)}_{s_{1}}{\left(EI_{z}\right)}_{s_{2}}\omega_{S_{3}}-L_{3}^{3}{\left(EI_{z}\right)}_{s_{2}}{\left(EI_{z}\right)}_{s_{3}}\omega_{S_{3}}+3L_{3}^{2}{\left(EI_{z}\right)}_{s_{1}}{\left(EI_{z}\right)}_{s_{2}}L_{1}\omega_{S_{1}}\\ & -3L_{3}^{2}{\left(EI_{z}\right)}_{s_{2}}{\left(EI_{z}\right)}_{s_{3}}L_{1}\omega_{S_{1}}+3L_{3}{\left(EI_{z}\right)}_{s_{1}}{\left(EI_{z}\right)}_{s_{2}}L_{1}^{2}\omega_{S_{1}}-3L_{3}{\left(EI_{z}\right)}_{s_{2}}{\left(EI_{z}\right)}_{s_{3}}L_{1}^{2}\omega_{S_{1}}\\ & -12L_{2}^{2}{\left(EI_{z}\right)}_{s_{1}}{\left(EI_{z}\right)}_{s_{3}}R_{0}+12L_{2}^{2}{\left(EI_{z}\right)}_{s_{2}}{\left(EI_{z}\right)}_{s_{3}}R_{0}-6L_{2}L_{3}{\left(EI_{z}\right)}_{s_{1}}{\left(EI_{z}\right)}_{s_{2}}R_{0}\\ & -6L_{2}L_{3}{\left(EI_{z}\right)}_{s_{1}}{\left(EI_{z}\right)}_{s_{3}}R_{0}+12L_{2}L_{3}{\left(EI_{z}\right)}_{s_{2}}{\left(EI_{z}\right)}_{s_{3}}R_{0}-12L_{2}{\left(EI_{z}\right)}_{s_{1}}{\left(EI_{z}\right)}_{s_{3}}L_{1}R_{0}\\ & +12L_{2}{\left(EI_{z}\right)}_{s_{2}}{\left(EI_{z}\right)}_{s_{3}}L_{1}R_{0}-3L_{3}^{2}{\left(EI_{z}\right)}_{s_{1}}{\left(EI_{z}\right)}_{s_{2}}R_{0}+3L_{3}^{2}{\left(EI_{z}\right)}_{s_{2}}{\left(EI_{z}\right)}_{s_{3}}R_{0}\\ & -6L_{3}{\left(EI_{z}\right)}_{s_{1}}{\left(EI_{z}\right)}_{s_{2}}L_{1}R_{0}+6L_{3}{\left(EI_{z}\right)}_{s_{2}}{\left(EI_{z}\right)}_{s_{3}}L_{1}R_{0}+12L_{2}{\left(EI_{z}\right)}_{s_{1}}{\left(EI_{z}\right)}_{s_{3}}M_{0}\\ & -12L_{2}{\left(EI_{z}\right)}_{s_{2}}{\left(EI_{z}\right)}_{s_{3}}M_{0}+6L_{3}{\left(EI_{z}\right)}_{s_{1}}{\left(EI_{z}\right)}_{s_{2}}M_{0}-6L_{3}{\left(EI_{z}\right)}_{s_{2}}{\left(EI_{z}\right)}_{s_{3}}M_{0}) \end{array}$$

(A8) $$\begin{array}{ll} C_{10}= & \frac{1}{24}\frac{1}{{\left(EI_{z}\right)}_{s_{1}}{\left(EI_{z}\right)}_{s_{2}}{\left(EI_{z}\right)}_{s_{3}}}(41L_{2}^{4}{\left(EI_{z}\right)}_{s_{1}}{\left(EI_{z}\right)}_{s_{3}}\omega_{S_{2}}+7L_{2}^{4}{\left(EI_{z}\right)}_{s_{1}}{\left(EI_{z}\right)}_{s_{3}}\omega_{S_{4}}\\ & -41L_{2}^{4}{\left(EI_{z}\right)}_{s_{2}}{\left(EI_{z}\right)}_{s_{3}}\omega_{S_{2}}-7L_{2}^{4}{\left(EI_{z}\right)}_{s_{2}}{\left(EI_{z}\right)}_{s_{3}}\omega_{S_{4}}+12L_{2}^{3}L_{3}{\left(EI_{z}\right)}_{s_{1}}{\left(EI_{z}\right)}_{s_{2}}\omega_{S_{2}}\\ & +60L_{2}^{3}L_{3}{\left(EI_{z}\right)}_{s_{1}}{\left(EI_{z}\right)}_{s_{3}}\omega_{S_{2}}+20L_{2}^{3}L_{3}{\left(EI_{z}\right)}_{s_{1}}{\left(EI_{z}\right)}_{s_{3}}\omega_{S_{3}}+4L_{2}^{3}L_{3}{\left(EI_{z}\right)}_{s_{1}}{\left(EI_{z}\right)}_{s_{3}}\omega_{S_{4}}\\ & -72L_{2}^{3}L_{3}{\left(EI_{z}\right)}_{s_{2}}{\left(EI_{z}\right)}_{s_{3}}\omega_{S_{2}}-20L_{2}^{3}L_{3}{\left(EI_{z}\right)}_{s_{2}}{\left(EI_{z}\right)}_{s_{3}}\omega_{S_{3}}-4L_{2}^{3}L_{3}{\left(EI_{z}\right)}_{s_{2}}{\left(EI_{z}\right)}_{s_{3}}\omega_{S_{4}}\\ & +64L_{2}^{3}{\left(EI_{z}\right)}_{s_{1}}{\left(EI_{z}\right)}_{s_{3}}L_{1}\omega_{S_{1}}+28L_{2}^{3}{\left(EI_{z}\right)}_{s_{1}}{\left(EI_{z}\right)}_{s_{3}}L_{1}\omega_{S_{2}}+4L_{2}^{3}{\left(EI_{z}\right)}_{s_{1}}{\left(EI_{z}\right)}_{s_{3}}L_{1}\omega_{S_{4}}\\ & -64L_{2}^{3}{\left(EI_{z}\right)}_{s_{2}}{\left(EI_{z}\right)}_{s_{3}}L_{1}\omega_{S_{1}}-28L_{2}^{3}{\left(EI_{z}\right)}_{s_{2}}{\left(EI_{z}\right)}_{s_{3}}L_{1}\omega_{S_{2}}-4L_{2}^{3}{\left(EI_{z}\right)}_{s_{2}}{\left(EI_{z}\right)}_{s_{3}}L_{1}\omega_{S_{4}}\\ & +18L_{2}^{2}L_{3}^{2}{\left(EI_{z}\right)}_{s_{1}}{\left(EI_{z}\right)}_{s_{2}}\omega_{S_{2}}+24L_{2}^{2}L_{3}^{2}{\left(EI_{z}\right)}_{s_{1}}{\left(EI_{z}\right)}_{s_{3}}\omega_{S_{2}}+30L_{2}^{2}L_{3}^{2}{\left(EI_{z}\right)}_{s_{1}}{\left(EI_{z}\right)}_{s_{3}}\omega_{S_{3}}\\ & -42L_{2}^{2}L_{3}^{2}{\left(EI_{z}\right)}_{s_{2}}{\left(EI_{z}\right)}_{s_{3}}\omega_{S_{2}}-30L_{2}^{2}L_{3}^{2}{\left(EI_{z}\right)}_{s_{2}}{\left(EI_{z}\right)}_{s_{3}}\omega_{S_{3}}+24L_{2}^{2}L_{3}{\left(EI_{z}\right)}_{s_{1}}{\left(EI_{z}\right)}_{s_{2}}L_{1}\omega_{S_{1}}\\ & +12L_{2}^{2}L_{3}{\left(EI_{z}\right)}_{s_{1}}{\left(EI_{z}\right)}_{s_{2}}L_{1}\omega_{S_{2}}+72L_{2}^{2}L_{3}{\left(EI_{z}\right)}_{s_{1}}{\left(EI_{z}\right)}_{s_{3}}L_{1}\omega_{S_{1}}+24L_{2}^{2}L_{3}{\left(EI_{z}\right)}_{s_{1}}{\left(EI_{z}\right)}_{s_{3}}L_{1}\omega_{S_{2}}\\ & +12L_{2}^{2}L_{3}{\left(EI_{z}\right)}_{s_{1}}{\left(EI_{z}\right)}_{s_{3}}L_{1}\omega_{S_{3}}-96L_{2}^{2}L_{3}{\left(EI_{z}\right)}_{s_{2}}{\left(EI_{z}\right)}_{s_{3}}L_{1}\omega_{S_{1}}-36L_{2}^{2}L_{3}{\left(EI_{z}\right)}_{s_{2}}{\left(EI_{z}\right)}_{s_{3}}L_{1}\omega_{S_{2}}\\ & +8L_{2}L_{3}^{3}{\left(EI_{z}\right)}_{s_{1}}{\left(EI_{z}\right)}_{s_{2}}\omega_{S_{2}}+4L_{2}L_{3}^{3}{\left(EI_{z}\right)}_{s_{1}}{\left(EI_{z}\right)}_{s_{2}}\omega_{S_{3}}+12L_{2}L_{3}^{3}{\left(EI_{z}\right)}_{s_{1}}{\left(EI_{z}\right)}_{s_{3}}\omega_{S_{3}}\\ & -12L_{2}^{2}L_{3}{\left(EI_{z}\right)}_{s_{2}}{\left(EI_{z}\right)}_{s_{3}}L_{1}\omega_{S_{3}}+72L_{2}^{2}{\left(EI_{z}\right)}_{s_{1}}{\left(EI_{z}\right)}_{s_{3}}L_{1}^{2}\omega_{S_{1}}-72L_{2}^{2}{\left(EI_{z}\right)}_{s_{2}}{\left(EI_{z}\right)}_{s_{3}}L_{1}^{2}\omega_{S_{1}}\\ & +12L_{2}L_{3}^{2}{\left(EI_{z}\right)}_{s_{1}}{\left(EI_{z}\right)}_{s_{2}}L_{1}\omega_{S_{2}}+24L_{2}L_{3}^{2}{\left(EI_{z}\right)}_{s_{1}}{\left(EI_{z}\right)}_{s_{3}}L_{1}\omega_{S_{1}}+12L_{2}L_{3}^{2}{\left(EI_{z}\right)}_{s_{1}}{\left(EI_{z}\right)}_{s_{3}}L_{1}\omega_{S_{3}}\\ & -48L_{2}L_{3}^{2}{\left(EI_{z}\right)}_{s_{2}}{\left(EI_{z}\right)}_{s_{3}}L_{1}\omega_{S_{1}}-12L_{2}L_{3}^{2}{\left(EI_{z}\right)}_{s_{2}}{\left(EI_{z}\right)}_{s_{3}}L_{1}\omega_{S_{2}}-12L_{2}L_{3}^{2}{\left(EI_{z}\right)}_{s_{2}}{\left(EI_{z}\right)}_{s_{3}}L_{1}\omega_{S_{3}}\\ & +36L_{2}L_{3}{\left(EI_{z}\right)}_{s_{1}}{\left(EI_{z}\right)}_{s_{2}}L_{1}^{2}\omega_{S_{1}}+36L_{2}L_{3}{\left(EI_{z}\right)}_{s_{1}}{\left(EI_{z}\right)}_{s_{3}}L_{1}^{2}\omega_{S_{1}}-72L_{2}L_{3}{\left(EI_{z}\right)}_{s_{2}}{\left(EI_{z}\right)}_{s_{3}}L_{1}^{2}\omega_{S_{1}}\\ & +24L_{2}{\left(EI_{z}\right)}_{s_{1}}{\left(EI_{z}\right)}_{s_{3}}L_{1}^{3}\omega_{S_{1}}-24L_{2}{\left(EI_{z}\right)}_{s_{2}}{\left(EI_{z}\right)}_{s_{3}}L_{1}^{3}\omega_{S_{1}}+3L_{3}^{4}{\left(EI_{z}\right)}_{s_{1}}{\left(EI_{z}\right)}_{s_{2}}\omega_{S_{3}}\\ & -3L_{3}^{4}{\left(EI_{z}\right)}_{s_{2}}{\left(EI_{z}\right)}_{s_{3}}\omega_{S_{3}}+8L_{3}^{3}{\left(EI_{z}\right)}_{s_{1}}{\left(EI_{z}\right)}_{s_{2}}L_{1}\omega_{S_{1}}+4L_{3}^{3}{\left(EI_{z}\right)}_{s_{1}}{\left(EI_{z}\right)}_{s_{2}}L_{1}\omega_{S_{3}}\\ & -18L_{3}^{2}{\left(EI_{z}\right)}_{s_{2}}{\left(EI_{z}\right)}_{s_{3}}L_{1}^{2}\omega_{S_{1}}+12L_{3}{\left(EI_{z}\right)}_{s_{1}}{\left(EI_{z}\right)}_{s_{2}}L_{1}^{3}\omega_{S_{1}}-12L_{3}{\left(EI_{z}\right)}_{s_{2}}{\left(EI_{z}\right)}_{s_{3}}L_{1}^{3}\omega_{S_{1}}\\ & -64L_{2}^{3}{\left(EI_{z}\right)}_{s_{1}}{\left(EI_{z}\right)}_{s_{3}}R_{0}+64L_{2}^{3}{\left(EI_{z}\right)}_{s_{2}}{\left(EI_{z}\right)}_{s_{3}}R_{0}-24L_{2}^{2}L_{3}{\left(EI_{z}\right)}_{s_{1}}{\left(EI_{z}\right)}_{s_{2}}R_{0}\\ & -72L_{2}^{2}L_{3}{\left(EI_{z}\right)}_{s_{1}}{\left(EI_{z}\right)}_{s_{3}}R_{0}+96L_{2}^{2}L_{3}{\left(EI_{z}\right)}_{s_{2}}{\left(EI_{z}\right)}_{s_{3}}R_{0}-96L_{2}^{2}{\left(EI_{z}\right)}_{s_{1}}{\left(EI_{z}\right)}_{s_{3}}L_{1}R_{0}\\ & -8L_{3}^{3}{\left(EI_{z}\right)}_{s_{2}}{\left(EI_{z}\right)}_{s_{3}}L_{1}\omega_{S_{1}}-4L_{3}^{3}{\left(EI_{z}\right)}_{s_{2}}{\left(EI_{z}\right)}_{s_{3}}L_{1}\omega_{S_{3}}+18L_{3}^{2}{\left(EI_{z}\right)}_{s_{1}}{\left(EI_{z}\right)}_{s_{2}}L_{1}^{2}\omega_{S_{1}}\\ & +96L_{2}^{2}{\left(EI_{z}\right)}_{s_{2}}{\left(EI_{z}\right)}_{s_{3}}L_{1}R_{0}-24L_{2}L_{3}^{2}{\left(EI_{z}\right)}_{s_{1}}{\left(EI_{z}\right)}_{s_{2}}R_{0}-24L_{2}L_{3}^{2}{\left(EI_{z}\right)}_{s_{1}}{\left(EI_{z}\right)}_{s_{3}}R_{0}\\ & +48L_{2}L_{3}^{2}{\left(EI_{z}\right)}_{s_{2}}{\left(EI_{z}\right)}_{s_{3}}R_{0}-48L_{2}L_{3}{\left(EI_{z}\right)}_{s_{1}}{\left(EI_{z}\right)}_{s_{2}}L_{1}R_{0}-48L_{2}L_{3}{\left(EI_{z}\right)}_{s_{1}}{\left(EI_{z}\right)}_{s_{3}}L_{1}R_{0}\\ & +96L_{2}L_{3}{\left(EI_{z}\right)}_{s_{2}}{\left(EI_{z}\right)}_{s_{3}}L_{1}R_{0}-48L_{2}{\left(EI_{z}\right)}_{s_{1}}{\left(EI_{z}\right)}_{s_{3}}L_{1}^{2}R_{0}+48L_{2}{\left(EI_{z}\right)}_{s_{2}}{\left(EI_{z}\right)}_{s_{3}}L_{1}^{2}R_{0}\\ & -8L_{3}^{3}{\left(EI_{z}\right)}_{s_{1}}{\left(EI_{z}\right)}_{s_{2}}R_{0}+8L_{3}^{3}{\left(EI_{z}\right)}_{s_{2}}{\left(EI_{z}\right)}_{s_{3}}R_{0}-24L_{3}^{2}{\left(EI_{z}\right)}_{s_{1}}{\left(EI_{z}\right)}_{s_{2}}L_{1}R_{0}\\ & +24L_{3}^{2}{\left(EI_{z}\right)}_{s_{2}}{\left(EI_{z}\right)}_{s_{3}}L_{1}R_{0}-24L_{3}{\left(EI_{z}\right)}_{s_{1}}{\left(EI_{z}\right)}_{s_{2}}L_{1}^{2}R_{0}+24L_{3}{\left(EI_{z}\right)}_{s_{2}}{\left(EI_{z}\right)}_{s_{3}}L_{1}^{2}R_{0}\\ & +48L_{2}^{2}{\left(EI_{z}\right)}_{s_{1}}{\left(EI_{z}\right)}_{s_{3}}M_{0}-48L_{2}^{2}{\left(EI_{z}\right)}_{s_{2}}{\left(EI_{z}\right)}_{s_{3}}M_{0}+24L_{2}L_{3}{\left(EI_{z}\right)}_{s_{1}}{\left(EI_{z}\right)}_{s_{2}}M_{0}\\ & +24L_{2}L_{3}{\left(EI_{z}\right)}_{s_{1}}{\left(EI_{z}\right)}_{s_{3}}M_{0}-48L_{2}L_{3}{\left(EI_{z}\right)}_{s_{2}}{\left(EI_{z}\right)}_{s_{3}}M_{0}+48L_{2}{\left(EI_{z}\right)}_{s_{1}}{\left(EI_{z}\right)}_{s_{3}}L_{1}M_{0}\\ & -48L_{2}{\left(EI_{z}\right)}_{s_{2}}{\left(EI_{z}\right)}_{s_{3}}L_{1}M_{0}+12L_{3}^{2}{\left(EI_{z}\right)}_{s_{1}}{\left(EI_{z}\right)}_{s_{2}}M_{0}-12L_{3}^{2}{\left(EI_{z}\right)}_{s_{2}}{\left(EI_{z}\right)}_{s_{3}}M_{0}\\ & +24L_{3}{\left(EI_{z}\right)}_{s_{1}}{\left(EI_{z}\right)}_{s_{2}}L_{1}M_{0}-24L_{3}{\left(EI_{z}\right)}_{s_{2}}{\left(EI_{z}\right)}_{s_{3}}L_{1}M_{0}). \end{array}$$
